# Effectiveness of Key Biodiversity Areas in representing global avian diversity

**DOI:** 10.1111/cobi.70000

**Published:** 2025-03-03

**Authors:** Tom P. Lansley, Olivia Crowe, Stuart H. M. Butchart, David P. Edwards, Gavin H. Thomas

**Affiliations:** ^1^ School of Biosciences University of Sheffield Sheffield UK; ^2^ BirdLife International Cambridge UK; ^3^ Department of Plant Sciences and Conservation Research Institute University of Cambridge Cambridge UK

**Keywords:** area of habitat, area‐based conservation, biogeography, bird conservation, conservation prioritization, Key Biodiversity Areas, threatened species, áreas clave para la biodiversidad, área del hábitat, conservación basada en el área, conservación de aves, biogeografía, especie amenazada, priorización de la conservación

## Abstract

Key Biodiversity Areas (KBAs) are the largest and most complete network of significant sites for the global persistence of biodiversity. Although important sites for birds worldwide have been relatively well assessed, a key question is how effectively the global KBA network represents avian diversity. We identified bird species, orders, habitats, and geographic regions that are underrepresented by KBAs. Area of Habitat (AOH) maps for 10,517 terrestrial bird species were cropped and masked by the extent of each KBA. Almost all species had at least one part of their seasonal distribution in one or more KBAs. Twenty‐nine species had no habitat overlap with KBAs, and 1900 species had <8% of their AOH overlapping KBAs. Species with KBAs identified for them (5219 trigger species) had on average 2.6% greater representation of their AOH in KBAs than species that did not. The extent of species’ AOH represented by KBAs varied with region, habitat, and taxonomic group. Northern North America had the most underrepresented terrestrial bird species (up to 178 underrepresented species per 100 km^2^). Terrestrial bird species of tropical forests were 12.8% better represented in KBAs than expected by chance, whereas boreal and temperate forest species were less well represented than expected by chance (74.4% and 25.1%, respectively). Among avian orders, Anseriformes and Charadriiformes were underrepresented in KBAs (29.0% and 17.9%, respectively), whereas Trogoniformes and Psittaciformes were better represented (16.2% and 6.9%, respectively) than expected by chance. Bird species for potential KBA identification include marsh antwren (*Formicivora paludicola*) and Tabar pitta (*Erythropitta splendida*). These are mainly due to recent changes in species’ taxonomy or their International Union for Conservation of Nature Red List category. Identifying poorly represented species and where they occur highlights shortfalls where expansion of the network could bring conservation benefits.

## Abstract (es)

Eficacia de las áreas clave para la biodiversidad en la representación de la diversidad mundial de aves

**Resumen:**

Las áreas clave para la biodiversidad (ACB) son la mayor y más completa red de lugares significativos para la persistencia global de la biodiversidad. Aunque la evaluación de los lugares importantes para las aves de todo el mundo ha sido relativamente buena, una cuestión clave es la eficacia con la que la red mundial de ACB representa la diversidad de aves. Identificamos especies, órdenes, hábitats y regiones geográficas de aves que están infrarrepresentados en las ACB. Recortamos los mapas de área de hábitat (AH) de 10,517 especies de aves terrestres y los enmascaramos con la extensión de cada ACB. Casi todas las especies tuvieron al menos una parte de su distribución estacional en una o más ACB. El hábitat de 29 especies no se solapó con las ACB y 1,900 especies tenían <8% de su AH solapado con las ACB. Las especies identificadas en ACB (5,219 especies desencadenantes) tuvieron en promedio un 2.6% más de representación de su AH en las ACB que las especies que no las tenían. La extensión del AH de las especies representado por las ACB varió con la región, el hábitat y el grupo taxonómico. El norte de Norteamérica fue la región con más especies de aves terrestres infrarrepresentadas (hasta 178 especies infrarrepresentadas por cada 100 km^2^). Las especies de aves terrestres de los bosques tropicales estaban un 12.8% mejor representadas en las ACB de lo que esperaríamos por azar, mientras que las especies de los bosques boreales y templados estaban menos representadas de lo que esperaríamos por azar (74.4% y 25.1%, respectivamente). Entre los órdenes de aves, los Anseriformes y los Charadriiformes estuvieron infrarrepresentados en las ACB (29.0% y 17.9%, respectivamente), mientras que los Trogoniformes y los Psittaciformes estuvieron mejor representados (16.2% y 6.9%, respectivamente) de lo esperado por azar. Entre las especies de aves para la identificación potencial de ACB se encuentran *Formicivora paludicola* y la *Epittrythropitta splendida*. Esto se debe principalmente a cambios recientes en la taxonomía de las especies o en la categoría de la Lista Roja de la Unión Internacional para la Conservación de la Naturaleza. La identificación de las especies poco representadas y de los lugares donde se encuentran resalta las carencias a las que la ampliación de la red podría aportar beneficios para la conservación.

## INTRODUCTION

Biodiversity is rapidly declining globally, driven by a range of threats including deforestation, unsustainable exploitation, and invasive species (Butchart et al., 2012; IPBES, 2019), with suggestions that we are entering the sixth mass extinction event. Species loss and associated ecosystem degradation have wide societal impacts, making the conservation of biological resources paramount (IPBES, 2019; Maxwell et al., 2020). Key to averting a global mass extinction is conservation of important sites through a range of approaches to deliver positive conservation outcomes, from strictly protected areas to sustainably managed multiuse areas. To direct such conservation action, one needs to know where the most important sites for biodiversity are located (Brooks et al., 2006).

Biodiversity is not evenly distributed, meaning some areas have much greater diversity and endemism than others (Brooks et al., 2006; Grenyer et al., 2006; Mittermeier et al., 1998; Schröter & Remme, 2016). Systematic efforts to delineate such locations at the site scale began in the late 1970s with the identification of Birdlife International's Important Bird and Biodiversity Areas (IBAs) (Donald et al., 2019). Since its launch in 2005, the Alliance for Zero Extinction has identified a network of 853 sites holding the last remaining populations of one or more endangered or critically endangered species (Alliance for Zero Extinction, 2023). Key Biodiversity Areas (KBAs) act as umbrellas in these and other approaches. A global standard for the identification of KBAs was published in 2016 that contains criteria for identifying sites of significance for the global persistence of biodiversity. KBAs comprise the world's largest network of sites of biodiversity importance. There are 16,333 KBAs identified for a wide range of taxa, including vertebrates, invertebrates, and plants, and sites of significance for ecosystems and ecological integrity are now being added. The KBA program provides a globally standardized approach to bottom‐up nationally led identification of sites (IUCN, 2016). Identification of a site as a KBA does not imply any particular management or governance regime, but 19% of KBAs are completely covered by protected areas or other effective area‐based conservation measures (OECMs), and 42% are partially covered by such measures (IUCN, 2021).

The extent to which the KBA network is complete and comprehensive is unknown. Therefore, identifying and filling gaps is an urgent need. Some gaps are known. For example, KBA identification for birds is only just beginning in terrestrial New Guinea, a highly biodiverse region with many threatened and endemic species but with a very small ornithological and biodiversity conservation community (BirdLife International, 2023a). Identified solely for birds, IBAs continue to form the largest majority of sites in the KBA network (Moussy et al., 2022). Other gaps include underrepresented taxa and ecosystems. For example, <4% of KBAs have been identified for fish species, and a similar percentage have been identified for invertebrates. Only 27% of KBAs are in marine ecosystems, and <23% are in freshwater ecosystems (Key Biodiversity Areas Partnership, 2024; Miqueleiz et al., 2023). Such patterns reflect biases in the availability of knowledge (e.g., on the distribution and extinction risk) for these species and ecosystems. Increasing efforts to assess species in poorly known groups for the International Union for Conservation of Nature (IUCN) Red List and then to identify KBAs for these species are urgent priorities. Under the Kunming–Montreal Global Biodiversity Framework, the world's governments aim to conserve 30% of land and seas, especially areas of particular importance for biodiversity, through protected areas and other area‐based conservation measures by 2030 (CBD, 2022). The KBA program plays an important role in targeting expansion of protected and conserved areas to achieve this target due to its standardized and transparent approach to identifying sites of particular importance for biodiversity (Plumptre et al., 2024).

We tested how well the global KBA network captures the distributions of the world's birds. We focused on birds because KBAs for birds have been identified worldwide in virtually all countries and conservation efforts for birds typically benefit many other species (Hawkes et al., 2019; Pakkala et al., 2003; Roberge et al., 2008). A total of 13,655 KBAs have been identified for birds, approximately two thirds of which are identified for one or more threatened species, half for congregatory species, and one third for geographically restricted species (Key Biodiversity Areas Partnership, 2024). Studies of conservation gaps at broad scale typically use extent of occurrence maps, which overestimate occupancy. We measured the overlap of the global KBA network with Area of Habitat (AOH) maps for terrestrial bird species. This allowed a fine‐grained examination of the effectiveness of KBAs. We then explored gaps in the KBA network for birds by identifying species that have no or low overlap between their AOH and KBAs; taxonomic orders, habitats, and geographic regions of underrepresented species; and level of representation for species in different IUCN Red List categories of extinction risk, with a focus on underrepresented threatened species. We aimed to inform efforts to update and strengthen the global network of important sites for the world's birds.

## METHODS

### Data

We used 11,985 AOH maps for 10,517 terrestrial bird species (Brooks et al., 2019; Lumbierres, Dahal, Di Marco, et al., 2022). Of 1484 migratory species included, breeding and nonbreeding AOH maps were available for 1479 and 1471 respectively. Breeding and nonbreeding maps were included in analyses separately due to the importance of representation in KBAs for both seasonal ranges. Not all migratory species had breeding and nonbreeding AOH maps available due to lack of data, meaning AOH maps could not be created for those missing ranges. AOH maps aim to represent the distribution of habitat within a species’ range (Brooks et al., 2019; Dahal et al., 2022; Lumbierres, Dahal, Di Marco, et al., 2022). AOH maps can improve the accuracy of conservation planning and actions, aid in monitoring habitat loss and fragmentation, and help in assessing species potential distributions and extinction risk (Lumbierres, Dahal, Soria, et al., 2022).

In addition to the 11,985 AOH maps used, we used range maps for 158 terrestrial bird species whose AOH maps were not usable due to inaccuracies such as those showing no suitable habitat was present. We also used range maps rather than AOH maps for some analyses to reduce computational demands (see “Evaluating representation of species groups in KBAs”). Range maps were obtained from BirdLife International and Handbook of the Birds of the World (2022). We used a shapefile of the boundaries of 16,012 KBAs (Appendix S1; BirdLife International, 2022). KBAs are identified for 13,837 species, including 5994 bird species. Our data set of terrestrial bird AOH maps includes 5219 of these species. Additional data in our analyses included taxonomic data (order, family, and year of taxonomic split if applicable), IUCN Red List category and year first threatened (if appropriate), habitat preferences, and whether the species currently has any KBAs identified for it, is range restricted, or is congregatory, all acquired from BirdLife International (2023b).

### Overlap of species’ AOH with KBAs

All analyses and data manipulation were conducted in RStudio (Posit Team, 2022). The KBA polygons were transformed to match the Behrmann equal area cylindrical projection of the AOH maps to allow analyses involving both. Given that the polygons of some KBAs overlap, they were first dissolved to remove overlaps. Some polygons with invalid, self‐intersecting geometries were solved using the sf package (Pebesma, 2018). However, 9 KBA polygons were not solved, with their geometries remaining invalid, and were removed from further analyses.

We created a data matrix with each combination of KBA and AOH map (191,795,955 combinations) (Lansley et al., 2024). The matrix included the area of habitat in the AOH map that was in each KBA. Each AOH map was cropped and masked by the extent of the KBA file with packages raster (Hijmans, 2022) and rgis (Rodriguez‐Sanchez, 2020). The matrix also included the name of the AOH (identification number) and the name of the KBA (identification number). Other variables included were the extent of each KBA (extracted from the KBA shapefile); area of the masked AOH map; percentage of habitat in the AOH that overlapped each KBA; and the country, region, and IBA status. The IUCN Red List category and other variables from additional taxonomic (BirdLife International, 2023c) and habitat data were added to the data matrix to assess gaps and biases in KBA coverage. Habitat data consisted of all habitats that each species lives in and the importance of each habitat (i.e., “suitable” or “major” [BirdLife International, 2023d]). We created a new variable, single important habitat, for which a species’ range has only one habitat classified as major. Summary statistics for each AOH map were also created.

We used the data matrix to identify species whose AOH maps did not overlap any KBAs, underrepresented species (those with <8% overlap with KBAs), and very underrepresented species (those with <5% overlap with KBAs). The threshold of 8% was chosen because KBAs cover 8.01% of Earth's terrestrial surface (Plumptre et al., 2023); therefore, ∼8% of species’ AOH would be overlapped by KBAs if species and KBAs were randomly distributed. The threshold of 5% was chosen to explore patterns for the least well‐covered species and to provide a test of sensitivity to the choice of threshold. Species maps with no overlap and very underrepresented threatened species were evaluated to determine whether they met KBA criteria and new KBAs could be identified.

We also explored coverage of AOH by protected areas with a shapefile of protected area boundaries from the World Database on Protected Areas (UNEP‐WCMC & IUCN, 2024). We focused on a subset of species: those with AOH maps that had no overlap with KBAs and species listed as threatened on the IUCN Red List that had <5% of their AOH overlapping KBAs. This analysis was conducted using the same methods as the analysis of overlap with KBAs. For all of these analyses, each AOH map was cropped and masked by all protected areas, and the area of overlap was calculated. Species referred to as underrepresented in KBAs included some for which one seasonal range was underrepresented in KBAs.

### Evaluating representation of species groups in KBAs

To test whether underrepresented or very underrepresented species in KBAs were biased toward particular habitats, families or orders, or threat statuses, we generated null distributions by selecting AOH maps from the set of 11,985. We did this to match the number of species maps that were underrepresented (<8%) in KBAs and to match the number of species maps that were very underrepresented (<5%) in KBAs. The null distributions consisted of 1000 random samples for each level of KBA representation. The probability of the observed number of underrepresented species maps from each taxonomic group of species or group of species associated with each habitat occurring by chance was determined using a binomial distribution. They showed the difference between the underrepresented species maps and the random samples in percentage of the total number of each group of AOH maps. A difference of 0 meant the low overlap group was not different from the random samples; a negative difference meant that category was represented less than expected by chance; and a positive difference meant that category was better represented than expected by chance. We referred to results as significant when they did not cross 0.

The methods described above allowed us to identify underrepresented groups of species. If a taxonomic group, habitat, or IUCN Red List category was identified as underrepresented in the KBA network, further investigation could be carried out to determine why and whether focused efforts could identify KBAs for such species. Statistics and figures are from the <8% overlap analysis unless stated, although many significant trends were similar in both analyses. The world map figures were created using grids from epm (Title et al., 2022), and all figures were plotted with ggplot2 (Wickham, 2016). Species richness grids were created using range maps with information, such as percent overlap of each AOH map with KBAs from the data matrix.

## RESULTS

**FIGURE 1:**
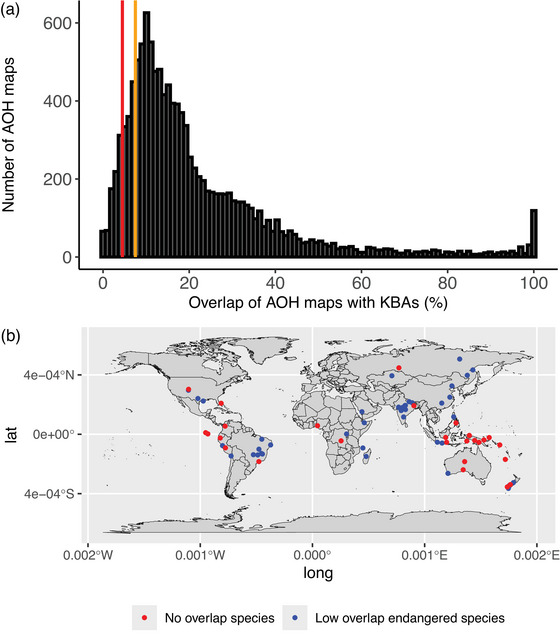
(a) Percentage of overlap between Area of Habitat (AOH) maps and Key Biodiversity Areas (KBAs) (red bar, species with <5% overlap; orange bar, species with < 8% overlap) and (b) location (centroid) of species’ AOH with no overlap with KBAs and of endangered and critically endangered species with <5% of their AOH overlapping KBAs.

Our results revealed overall high levels of species representation within KBAs but pointed to key shortfalls. Of the 11,985 AOH maps available for 10,517 terrestrial bird species (including separate maps for breeding and nonbreeding ranges of migratory species), the majority (10,488 species, 99.7%) overlapped KBAs. The majority of AOH maps (63.4%) had <20% overlap with KBAs, but 80 AOH maps had 100% overlap with KBAs. A total of 29 species had no overlap with any KBA. However, far more species had low overlap with KBAs: 970 AOH maps were very underrepresented (<5% coverage by KBAs) and 2190 were underrepresented (<8% coverage) (Figure 1a). For the maps that had no overlap, 2 represented breeding ranges (brown‐capped rosy finch [*Leucosticte australis*] and slender‐billed curlew [*Numenius tenuirostris*]), and the other 27 represented resident species (Appendices S2–S4). The 29 species included 7 critically endangered (of which 3 had no confirmed records for several decades), 4 endangered, and 7 vulnerable species. Many of these 29 species were recognized taxonomically, uplisted from least concern, or both since KBAs were identified within the countries in which they occur. Sixteen were first recognized taxonomically since 1998, and 3 others were reclassified from least concern to threatened or near threatened since then. They were therefore not eligible to trigger KBA criteria when most of the KBAs within their ranges were last assessed. Bird species’ AOH maps with KBAs identified for them (trigger species) had greater representation in KBAs (mean overlap = 24.2%) than species that did not have identified KBAs (mean overlap = 21.6%).

### Distribution of underrepresented species

There was marked geographic variation in the extent to which KBAs overlapped species’ AOH maps. A large proportion of the 29 species maps that did not overlap any KBAs were on islands (18 of 29), including Tabar pitta (*Erythropitta splendida*), Tabar Islands, Papua New Guinea, and the global biodiversity hotspots of the Andes and Southeast Asia supported many of the remainder. In the rest of Asia, Africa, and North America, there were relatively fewer species maps with no KBA overlap, whereas AOH maps of all species in Europe overlapped at least one KBA (Figure 1b).

**FIGURE 2:**
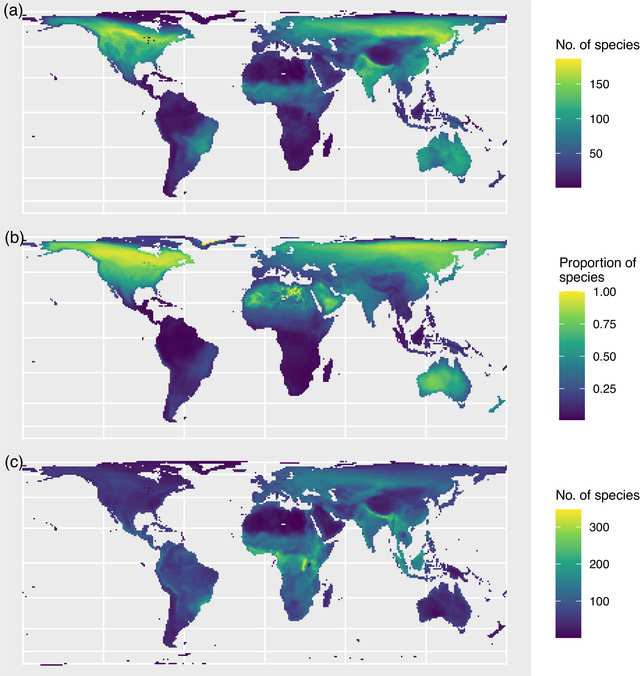
(a) Number of underrepresented species with <8% of their area of habitat (AOH) overlapping with Key Biodiversity Areas (KBAs) (*n* = 1874), (b) proportion of species (*n* = 10,969) with <8% of their AOH overlapping KBAs, and (c) number of species for which KBAs have been identified (*n* = 5195).

Underrepresented species maps were concentrated in northern North America (up to 178 species maps per 100‐km cell), India, Russia, China, and Australia (Figure 2a). Such species maps comprised a higher proportion of all species maps occurring in each 100‐km grid cell in northern North America, northeastern Asia, and southeastern Australia (Figure 2b). The patterns for very underrepresented species were similar (Appendices S6 & S7); North America had the greatest proportion of very underrepresented species. Like many of the species that did not have any overlap between their AOH and KBAs, 403 species (with 454 underrepresented AOH maps) have been recognized taxonomically by BirdLife International since 1998, including 197 species (212 AOH maps) that were very underrepresented. Such species were concentrated in central Africa, southern Asia, parts of South America, eastern Europe, and western Asia (Figure 2c). North America, northern Africa, and Australia had fewer of these species.

### Habitat representation in KBAs

**FIGURE 3:**
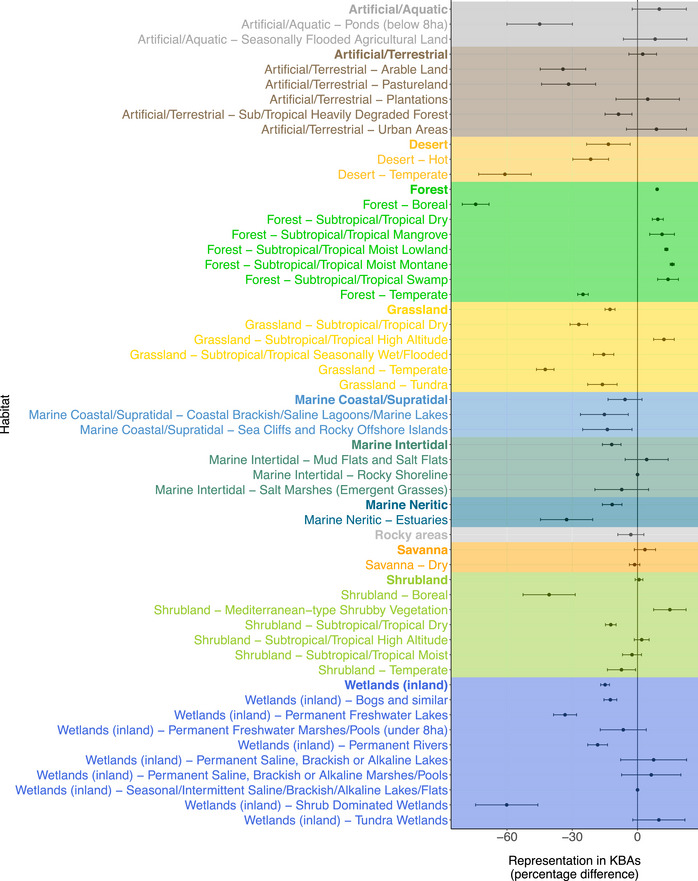
The difference between the number of underrepresented species (i.e., species with <8% of their area of habitat [AOH] overlapping with Key Biodiversity Areas [KBAs]) (*n* = 2190) with only one important habitat (level 1 habitats shown in bold [IUCN, 2024]) and 1000 random samples of 2190 AOH maps from the full set of 11,985 terrestrial avian AOH maps as a percentage of the total number of AOH maps in that habitat (i.e., the difference between observed number of species underrepresented by KBAs and the number expected by chance by habitat) (difference of 0, number of underrepresented species not different from random samples; negative difference [e.g., boreal forest, 74.4%], category represented less than expected by chance; positive difference [e.g., forest, 9.0%], category better represented than expected).

Species representation within KBAs was also highly dependent on habitat. Species for which forest was of major importance were significantly better represented in KBAs than would be expected by chance (9.0% more AOH maps with ≥8% overlap with KBAs than expected) (Figure 3; Appendices S8–S10), whereas species associated with 5 other broad (level 1) habitat types (IUCN, 2024), including inland wetlands and grassland, were significantly less well represented than expected by chance (by 14.9% and 12.6%, respectively). Among finer‐scale (level 2 habitat classes for species, tropical and subtropical forest (moist lowland, moist montane, and to a lesser extent dry forest) species were better represented than expected (by 13.3%, 16.0%, and 9.3% respectively), whereas boreal and temperate forest species were represented less than expected by chance (by 74.4% and 25.1%, respectively). There were no threatened bird species with boreal forest as their only important habitat (such habitat specialists comprise 35 least concern and 4 near‐threatened species). Of 240 species maps with temperate forest as their only important habitat, 6 were listed as critically endangered, 5 as endangered, and 20 as vulnerable. Other habitats supported an insufficient number of species for a meaningful assessment of representation, including shrub‐dominated wetlands and temperate desert (3 and 6 AOH maps, respectively). Ten finer‐scale habitats had no underrepresented species, such as moist savanna (8 AOH maps).

### Taxonomic group representation in KBAs

**FIGURE 4:**
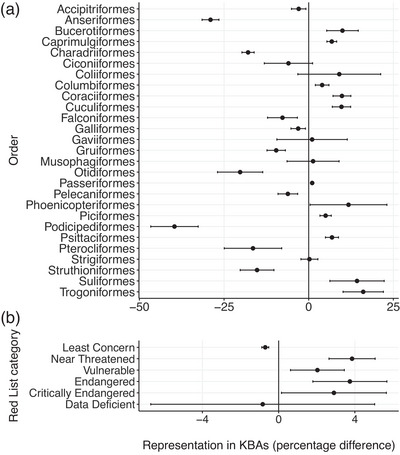
The difference between the number of underrepresented species (the 2190 Area of Habitat [AOH] maps with <8% of their suitable habitat overlapping Key Biodiversity Areas [KBAs]) and 1000 random samples of 2190 AOH maps from the full set of 11,985 terrestrial avian AOH maps as a percentage of the total number of AOH maps in that (a) order and (b) International Union for Conservation of Nature Red List category (difference of 0, number of underrepresented species not different from random samples; negative difference [e.g., Anseriformes], category represented less than expected by chance; positive difference [e.g., Bucerotiformes], category better represented than expected).

Although some bird orders and families were well represented in the KBA network, others were less well represented than expected by chance alone. Eleven bird orders were significantly less well represented in KBAs than expected by chance, including Anseriformes (29.0% more AOH maps with <8% overlap with KBAs than expected) and Charadriiformes (by 17.9%) (Figure 4a; Appendices S11–S13). Conversely, 11 were significantly better represented in KBAs than expected by chance, including Trogoniformes (by 16.2%) and Psittaciformes (by 6.9%). No AOH maps from 6 orders, all with small sample sizes (maximum 8 AOH maps), were underrepresented. Similarly, no AOH maps from 85 families (75 with a sample size of <10 AOH maps) were underrepresented (Appendices S14–S17). Of these families, Tityridae and Ramphastidae had the most AOH maps (51 and 50, respectively) and were therefore considered well represented in KBAs. Families with better representation in KBAs than would be expected by chance included Dicaeidae (by 16.7%) and Thamnophilidae (by 12.0%). Others had significantly less representation than expected by chance, such as Emberizidae (by 31.2%) and Anatidae (by 29.5%).

### Variation in species’ representation in KBAs by IUCN Red List category

Threatened (i.e., vulnerable, endangered, and critically endangered species) and near‐threatened species were significantly better represented in KBAs than expected by chance, which is unsurprising given sites supporting threatened species may qualify as KBAs under KBA criterion A. The percentage of AOH maps that were underrepresented in KBAs was 18.3%. All threatened and near‐threatened IUCN Red List categories had fewer than 18.3% underrepresented AOH maps (range: 14.4–16.2%), which showed that they were better represented than the total percentage. Similarly, least concern species were represented in KBAs significantly less than expected by chance (Figure 4b; Appendices S18–S20). Among underrepresented (<8% overlap) and very underrepresented (<5% overlap) species, the only category that showed consistent results was near threatened, which was better represented than expected by chance. All categories had mean percent differences from the random samples of <4%.

There were 111 threatened (18 critically endangered, 32 endangered, and 61 vulnerable) species that were very underrepresented in KBAs in at least one part of their seasonal distribution (Figure 1b; Appendix S5). Of these species, 76 were resident, 6 were migratory (breeding and nonbreeding ranges had <5% of their AOH overlapping KBAs), 19 were very underrepresented in only their breeding range, and 10 were very underrepresented in only their nonbreeding range. Of the very underrepresented threatened species maps and the species maps with no overlap with KBAs (122), 21 had no representation in protected areas (Appendix S21). Very underrepresented species were particularly concentrated in New Zealand, Brazil, India, and eastern Asia. Twenty‐four of these 111 species did not have any KBAs identified for them. For these 24, and almost half of the other 87 species that did have at least one KBA identified, their low representation was explained by KBA inventories not being updated since the taxa were recognized as species, by their uplisting to threatened on the IUCN Red List or to more threatened categories, or by terrestrial KBAs not having been identified within the species’ range (Appendix S5). Some of these species could meet KBA criteria in existing KBAs or in areas not yet identified as KBAs (e.g., Sharpe's lark [*Mirafra sharpii*]).

## DISCUSSION

Our results showed that the global KBA network is remarkably efficient at representing the global diversity of terrestrial birds. The global network of KBAs included habitat for the vast majority of the world's birds: only 29 of 11,985 species maps (0.24%) relating to 10,517 terrestrial bird species (0.28%) had no overlap with any KBA (and 2 of these species had nonbreeding AOH in KBAs). These 29 species were largely small‐ranged species (mean range of 31,460 km^2^; 26 species had ranges smaller than 8500 km^2^) that have been recognized taxonomically, uplisted from least concern, or both since the KBAs were identified in the countries in which they occur (16 were first recognized since 1998 and 5 more were first listed as threatened or near threatened since then). Threatened and near‐threatened species were better represented in KBAs than least concern and data‐deficient species, as would be expected given the KBA criteria.

### Underrepresentation of species in KBAs

A key reason for some species being very underrepresented or not represented at all in KBAs was because they were not recognized taxonomically or not considered threatened at the time when KBAs were identified in the countries within their range, or both. This explained 19 of 29 species maps with no overlap with KBAs and 67 of 111 very underrepresented threatened species. Efforts are urgently needed to determine whether any locations qualify as KBAs for these species and if so to delineate and propose these sites as KBAs. For other poorly represented species, additional sites are likely to qualify as KBAs, but further surveys are required. For example, the breeding range of streaked reed‐warbler (*Acrocephalus sorghophilus*) is unknown, and there has been only one record (unconfirmed) in the nonbreeding range since 2015 (BirdLife International, 2023b; Fregin et al., 2009). Other species are not particularly well suited to site‐based conservation owing to their low population densities across very large ranges, including threatened species such as Blakiston's fish owl (*Bubo blakistoni*) and white‐eared night heron (*Oroanassa magnifica*), although further surveys may detect additional sites qualifying as KBAs (BirdLife International, 2023b; Slaght et al., 2018). For other underrepresented species that are relatively easy to detect (e.g., *Grus americana*), we are confident that the key sites that would qualify as KBAs have already been identified as such (BirdLife International, 2023b). In some cases, it is likely that sufficient data already exist to propose sites as KBAs (see “Conservation Action” below).

Some migratory species were underrepresented in one part of their range but much better represented in another. For example, far eastern curlew (*Numenius madagascariensis*) had only 2.2% of its breeding AOH overlapping KBAs but 15.5% of its nonbreeding AOH overlapping KBAs. This is because it breeds at densities too low to trigger KBA identification but congregates at key locations in the nonbreeding season. For such species, broadscale policy responses targeting land use and land management are required to safeguard the breeding distribution as are targeted site‐scale efforts to protect and effectively manage important sites used during migration and in the nonbreeding season.

In some cases, low coverage of species’ area of habitat by KBAs may have resulted from errors in the underlying data sets. Some KBA boundaries may be inaccurate due to digitization errors or misalignment; a small number (312) of KBAs lacked digital boundaries and were therefore excluded from our analyses. Errors in the AOH maps may have originated from errors in range maps, documented habitat and elevation preferences, and land‐cover maps (Brooks et al., 2019; Lumbierres, Dahal, Soria, et al., 2022). In some cases (e.g., Prigogine's greenbul [*Chlorocichla prigoginei*]), we found no or very low overlap between species’ AOH maps and the KBAs identified for them, suggesting errors in the data. For some species, there were clear mismatches between their AOH map boundary and those of the KBAs identified for them that could not be fully explained. For example, the boundary of the Karthala Mountains KBA, which is based on an old assessment, did not fully reflect the distribution of *Dicrurus fuscipennis*, an endemic of the Comoro Archipelago. The KBA and AOH data sets (and the underlying range maps and habitat and elevation data) are continually being improved, so their accuracy should increase in future. A mechanism to facilitate feedback between the KBA identification and IUCN Red List assessment processes would improve the quality of the respective data sets and thus benefit conservation.

### Patterns in underrepresented species

Underrepresented species occurred in a wide range of locations, although there were moderate concentrations in the boreal forests of northern North America and northeastern Asia (Figure 1b). These are areas where the majority of bird species are not threatened, have large distributions, and do not congregate (with the exception of waterbirds), so there are fewer species for which KBAs can be potentially identified, and relatively few sites support a sufficient proportion of the global population to qualify as KBAs (e.g., KBAs for birds cover just 2% of the terrestrial extent of Canada [Key Biodiversity Areas Partnership, 2024]). The lower representation of boreal forest species in KBAs therefore reflected the fact that site‐scale conservation is less appropriate for this extremely extensive habitat and the typically common and widely distributed species that occur in it (Imbeau et al., 2001; Wells et al., 2020). A reassessment of the KBA network in Canada is currently underway, with 729 candidate sites identified already (across all taxonomic groups and KBA criteria), so KBA coverage of bird species in boreal forest is likely to increase (KBA Canada, 2024). Alongside site conservation, landscape‐scale policies are needed in this biome to retain remaining intact habitat, restore degraded forest, reduce fragmentation, and ensure that forestry is managed sustainably. In tropical and subtropical forest, fewer species were underrepresented in KBAs than expected by chance, likely because these species‐rich habitats contain many range‐restricted and threatened species for which KBAs have been identified.

We found considerable overlap between the sets of underrepresented species we identified. For example, underrepresented species were concentrated in northern North America and Russia (Figure 2; Appendices S6 & S7), which contain considerable extents of boreal forest (Gauthier et al., 2015) (Figure 3) and are important for supporting species of Emberizidae (in Asia) and Parulidae (in North America). Similarly, species from the underrepresented orders Anseriformes, Charadriiformes, and Podicipediformes (Figure 4) predominantly live in wetlands (Chopra et al., 2013), which is an underrepresented habitat. For example, scaly‐sided merganser (*Mergus squamatus*), which has <1% of its AOH overlapping KBAs, is endangered, lives in northeastern Asia, is in the family Anatidae, and has inland wetlands—permanent rivers—as its only habitat of importance in the nonbreeding season. Some species from these orders that are congregatory and that occur predominantly in wetland KBAs may be better represented than indicated here because they have been assessed using other KBA assessment parameters, such as number of mature individuals, derived from count data rather than area‐relevant parameters (IUCN, 2016).

### Conservation action

KBAs are identified nationally through standardized criteria with quantitative thresholds. The process is typically managed by KBA National Coordination Groups who organize the identification process and support the integration of KBAs in local and national policy (Lim et al., 2023). Our results can assist with the identification of species that should be given particular attention when undertaking KBA assessments because they have zero or low KBA coverage. For example, the distribution of the critically endangered marsh antwren (*Formicivora paludicola*) in the Brazilian Atlantic Forest did not overlap any KBAs because it was not assessed by the IUCN until 2016 (following taxonomic revision leading to its recognition as full species), which was subsequent to the identification of KBAs in Brazil. However, given its extremely small distribution, it is likely that one or more locations support a sufficiently large proportion of the global population to qualify as a KBA. Similarly, 2 vulnerable species that occur only on Genovesa Island, Galapagos, Ecuador, were highlighted by our results as lacking any KBAs for similar reasons (they were first assessed for the IUCN Red List following taxonomic revision subsequent to the identification of KBAs in Ecuador [Appendix S4]). However, independent of our work, a reassessment of KBAs in Ecuador published in June 2024 led to their addition as a qualifying species for the Galapagos Archipelago and Marine Reserve KBA (Key Biodiversity Areas Partnership, 2024). The vulnerable Tabar pitta (*E. splendida*) in Papua New Guinea is an example of a species with no representation in KBAs or protected areas. Other species may occur in one or more KBAs but lack any overlap with protected areas. The KBAs they occur in are therefore particular priorities for protection. For example, the endangered Sharpe's lark (*M. sharpii*) in Somalia and the vulnerable Naga wren‐babbler (*Spelaeornis chocolatinus*) in India were very underrepresented in KBAs and had no representation in protected areas.

Our results support efforts to update and expand KBA inventories to make these networks more comprehensive. In particular, these efforts should focus on threatened species with no or a low proportion of their habitat within KBAs (Appendices S4 & S5), especially those recognized taxonomically or uplisted from least concern since KBAs were last updated within their countries of occurrence. More generally, our results highlight the need for regular reassessment of KBA networks to ensure they reflect the latest information on taxonomy, extinction risk, occurrence, and abundance of bird species.

The KBA network provides extensive coverage of the distribution of the world's birds and is useful for targeting efforts to safeguard and effectively conserve birds worldwide, as well as playing an important part in global conservation planning. However, additional efforts are needed to update the inventory, especially for underrepresented species. Such work sits alongside efforts to safeguard existing KBAs, in particular to expand national protected area networks, recognize OECMs, and secure effective conservation action on the ground, especially for globally threatened species. Such efforts will deliver wider benefits to biodiversity, given the occurrence of many other species of concern in sites of importance identified for birds and the benefits to people that these locations provide (BirdLife International, 2014, 2024; Donald et al., 2019; Waliczky et al., 2019).

The KBA network is important for informing policy and practice and is highly relevant to the goals and targets of the Kunming–Montreal Global Biodiversity Framework. For example, Target 3 calls for the protection and conservation of 30% of land and seas, especially “areas of particular importance for biodiversity,” and references KBAs as the most comprehensive, systematically identified, global network of such sites. Similarly, Goal A and Target 4 call for human‐driven extinctions of known threatened species to be halted. KBAs identified under criterion A1e hold the last remaining populations of one or more critically endangered or endangered species (IUCN, 2016). Their conservation is therefore essential to prevent extinctions. We highlighted which threatened species are currently not well represented in the KBA network. This information allowed us to identify priorities for updating national KBA assessments to ensure that protected area expansion is effectively targeted to the most important locations for the persistence of biodiversity.

## Supporting information



Supporting Information
