# Supplementary material for: Effectiveness of Key Biodiversity Areas in representing global avian diversity
Source: Conserv Biol. 2025 Mar 3;39(4):e70000. doi: 10.1111/cobi.70000 (PMC12309649; doi:10.1111/cobi.70000)
Supplement: Supplementary file 1 — Supporting Information [file COBI-39-e70000-s001.docx]

# How effectively do Key Biodiversity Areas represent global avian diversity?

# Supporting Information


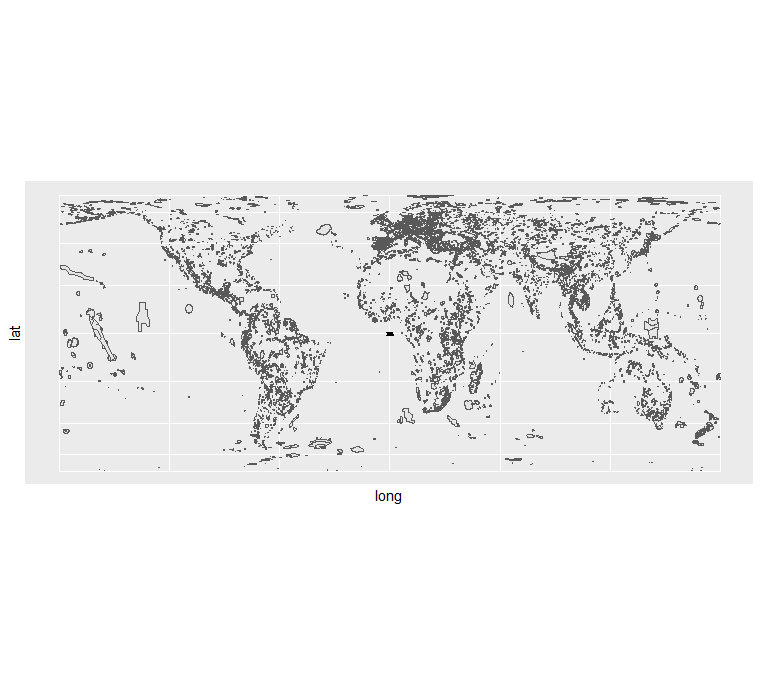


Appendix S1. Polygons of Key Biodiversity Areas (n = 16,003).

## Appendix S2: Description of critically endangered species with no KBA overlap

The critically endangered species with no overlap with KBAs are chestnut-capped Piha (*[Lipaugus weberi](http://datazone.birdlife.org/species/factsheet/22729457)*) which has an estimated population of <250 in the Colombian Andes and is likely still declining with rapid deforestation and fragmentation (Cuervo, 2014). Marsh Antwren (*[Formicivora paludicola](http://datazone.birdlife.org/species/factsheet/103657434)*) has an estimated population of 150-700 mature individuals, east of Sao Paulo, Brazil (De Camargo *et al.*, 2015). They have a severely fragmented population, including three subpopulations with considerable genetic differentiation. Their habitat is becoming less suitable due to human development and an invasive grass species. Bahama nuthatch ([*Sitta insularis*](http://datazone.birdlife.org/species/factsheet/103881687)) is only found on Grand Bahama Island, the Bahamas and has an estimated breeding extent of 80 km^2^. Its population has been in severe decline (over 90% in the last ten years) due to forest loss, invasive species, and severe hurricanes in 2016 and 2019. It is possible that this species has gone extinct in recent years, with no recent confirmed recordings (Bell, 2019; Parks, 2023; Pereira *et al.*, 2023). Slender-billed curlew ([*Numenius tenuirostris*](http://datazone.birdlife.org/species/factsheet/22693185)) overlaps many KBAs in its nonbreeding range but none in its small known breeding range in Russia. The last confirmed record of this species was in 2001 (BirdLife International, 2023). Many of these suggestions are due to species that are newly recognized, mostly by taxonomic splits, after the country it is present in identified their KBAs and IBAs.


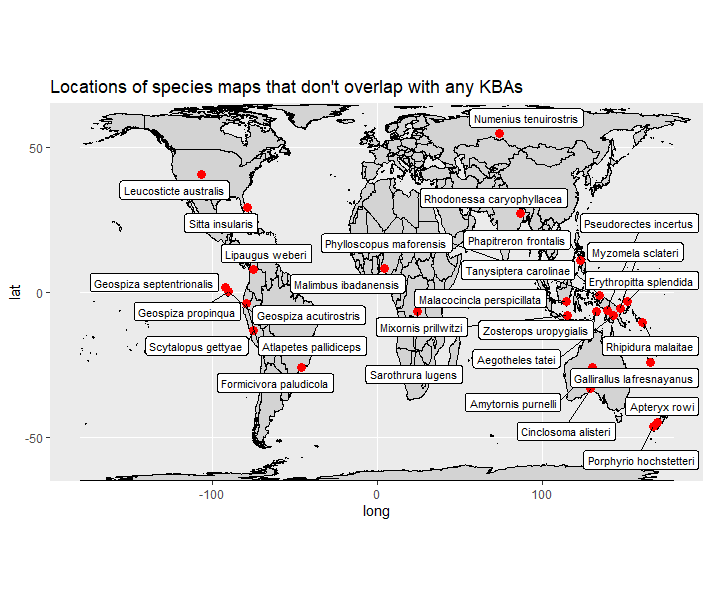


Appendix S3. Location (centroid) of species maps that do not overlap with any Key Biodiversity Areas (KBAs), with labels.

Appendix S4. Species maps that do not overlap with Key Biodiversity Areas (KBAs). Area of Habitat maps show area of suitable habitat, range maps (identified with an asterisk *) show the size of their full range.

| Species | Range | Order | Family | Red List category | Year recognized by BirdLife | Year first recognized as threatened | Area of suitable habitat  (km^2^) |
| --- | --- | --- | --- | --- | --- | --- | --- |
| [*Erythropitta splendida*](http://datazone.birdlife.org/species/factsheet/103656470) | Resident | Passeriformes | Pittidae | VU | 2016 | 2016 | 262.24 |
| [*Formicivora paludicola*](http://datazone.birdlife.org/species/factsheet/103657434) | Resident | Passeriformes | Thamnophilidae | CR | 2016 | 2016 | 29.7 |
| [*Scytalopus gettyae*](http://datazone.birdlife.org/species/factsheet/103660951) | Resident | Passeriformes | Rhinocryptidae | NT | 2016 | 2016 | 533.59 |
| [*Cinclosoma alisteri*](http://datazone.birdlife.org/species/factsheet/103693230) | Resident | Passeriformes | Cinclosomatidae | LC | 2016 | NA | 36902.34 |
| [*Geospiza acutirostris*](http://datazone.birdlife.org/species/factsheet/103815404) | Resident | Passeriformes | Thraupidae | VU | 2016 | 2016 | 3.59 |
| [*Geospiza propinqua*](http://datazone.birdlife.org/species/factsheet/103815544) | Resident | Passeriformes | Thraupidae | VU | 2016 | 2016 | 3.59 |
| [*Sitta insularis*](http://datazone.birdlife.org/species/factsheet/103881687) | Resident | Passeriformes | Sittidae | CR | 2016 | 2016 | 6.24 |
| [*Mixornis prillwitzi*](http://datazone.birdlife.org/species/factsheet/103894733) | Resident | Passeriformes | Timaliidae | VU | 2016 | 2016 | 144.68 |
| [*Rhodonessa caryophyllacea*](http://datazone.birdlife.org/species/factsheet/22680344) | Resident | Anseriformes | Anatidae | CR | 1988 | 1988 | 0.97 |
| [*Tanysiptera carolinae*](http://datazone.birdlife.org/species/factsheet/22683592) | Resident | Coraciiformes | Alcedinidae | NT | 1988 | 2000 | 288.02 |
| [*Sarothrura lugens*](http://datazone.birdlife.org/species/factsheet/22692227) | Resident | Gruiformes | Rallidae | LC | 1988 | NA | 16034.67 |
| [*Gallirallus lafresnayanus*](http://datazone.birdlife.org/species/factsheet/22692388) | Resident | Gruiformes | Rallidae | CR | 1988 | 1988 | 0.96 |
| [*Porphyrio hochstetteri*](http://datazone.birdlife.org/species/factsheet/22692808) | Resident | Gruiformes | Rallidae | EN | 1996 | 1996 | 397.92 |
| [*Myzomela sclateri*](http://datazone.birdlife.org/species/factsheet/22703874) | Resident | Passeriformes | Meliphagidae | LC | 1988 | NA | 963 |
| [*Pseudorectes incertus*](http://datazone.birdlife.org/species/factsheet/22705579) | Resident | Passeriformes | Pachycephalidae | NT | 1988 | 2000 | 595.12 |
| [*Rhipidura malaitae*](http://datazone.birdlife.org/species/factsheet/22706910) | Resident | Passeriformes | Rhipiduridae | VU | 1988 | 1988 | 4.88 |
| [*Zosterops uropygialis*](http://datazone.birdlife.org/species/factsheet/22714103) | Resident | Passeriformes | Zosteropidae | NT | 1988 | 1988 | 336 |
| [*Malimbus ibadanensis*](http://datazone.birdlife.org/species/factsheet/22719091) | Resident | Passeriformes | Ploceidae | EN | 1988 | 1988 | 1647.04 |
| [*Atlapetes pallidiceps*](http://datazone.birdlife.org/species/factsheet/22721487) | Resident | Passeriformes | Passerellidae | EN | 1988 | 1988 | 0.04 |
| [*Aegotheles tatei*](http://datazone.birdlife.org/species/factsheet/22728459) | Resident | Caprimulgiformes | Aegothelidae | DD | 2000 | 2000 | 3095.4 |
| [*Leucosticte australis*](http://datazone.birdlife.org/species/factsheet/22728985) | Breeding | Passeriformes | Fringillidae | EN | 1988 | 2018 | 5245.23 |
| [*Lipaugus weberi*](http://datazone.birdlife.org/species/factsheet/22729457) | Resident | Passeriformes | Cotingidae | CR | 2002 | 2002 | 22.76 |
| [*Apteryx rowi*](http://datazone.birdlife.org/species/factsheet/22732871) | Resident | Struthioniformes | Apterygidae | VU | 2014 | 2014 | 164.37 |
| [*Amytornis purnelli*](http://datazone.birdlife.org/species/factsheet/22735659) | Resident | Passeriformes | Maluridae | LC | 2009 | NA | 653179.1 |
| [*Phapitreron frontalis*](http://datazone.birdlife.org/species/factsheet/60543516) | Resident | Columbiformes | Columbidae | CR | 2014 | 2014 | 0.59 |
| [*Geospiza septentrionalis*](http://datazone.birdlife.org/species/factsheet/103815245)*** | Resident | Passeriformes | Thraupidae | VU | 2016 | 2016 | <128.66* |
| [*Phylloscopus maforensis*](http://datazone.birdlife.org/species/factsheet/103866023)*** | Resident | Passeriformes | Phylloscopidae | NT | 2016 | 2016 | <33820.48* |
| [*Numenius tenuirostris*](http://datazone.birdlife.org/species/factsheet/22693185)*** | Breeding | Charadriiformes | Scolopacidae | CR | 2016 | 2016 | <841218.6* |
| [*Malacocincla perspicillata*](http://datazone.birdlife.org/species/factsheet/22715827)*** | Resident | Passeriformes | Pellorneidae | DD | 2016 | NA | <97.21* |

Appendix S5. AOH maps of endangered and critically endangered species that are very under-represented by Key Biodiversity Areas (KBAs) (<5% of their suitable habitat overlaps with KBAs).

| Species | Range | Red List category | Year recognized by BirdLife | Year first recognized as threatened | Year last uplisted to EN/CR | Area of suitable habitat  (km^2^) | Percentage of suitable habitat that overlaps KBAs |
| --- | --- | --- | --- | --- | --- | --- | --- |
| *Malimbus ibadanensis* | Resident | EN | 1988 | 1988 | 1994 | 1647.04 | 0 |
| *Atlapetes pallidiceps* | Resident | EN | 1988 | 1988 | 1994 | 0.04 | 0 |
| *Lipaugus weberi* | Resident | CR | 2002 | 2002 | 2015 | 22.76 | 0 |
| *Numenius tenuirostris* | Breeding | CR | 1988 | 1988 | 1994 | AOH not available. | 0 |
| *Acrocephalus sorghophilus* | Resident | CR | 1988 | 1988 | 2022 | 1269.19 | 0.006303 |
| *Columbina cyanopis* | Resident | CR | 1988 | 1988 | 1994 | 168.24 | 0.820257 |
| *Charadrius obscurus* | Resident | CR | 2014 | 2014 | 2017 | 212.46 | 1.332015 |
| *Chlorocichla prigoginei* | Resident | EN | 1988 | 1988 | 2000 | 352.39 | 0.857005 |
| *Dicrurus fuscipennis* | Resident | EN | 1988 | 1988 | 1994 | 197.11 | 2.627974 |
| *Xenicus gilviventris* | Resident | EN | 1988 | 1994 | 2016 | 3969.24 | 0.293255 |
| *Mirafra sharpii* | Resident | EN | 1988 | 2016 | 2016 | 2506.75 | 0.898773 |
| *Poospiza rubecula* | Resident | EN | 1988 | 1988 | 1994 | 1268.25 | 3.01439 |
| *Mergus octosetaceus* | Resident | CR | 1988 | 1988 | 1994 | 3231.17 | 1.231442 |
| *Grus americana* | nonbreeding | EN | 1988 | 1988 | 1994 | 3971.25 | 2.276361 |
| *Rhinoptilus bitorquatus* | Resident | CR | 1988 | 1988 | 2000 | 3050.68 | 3.735888 |
| *Hymenolaimus malacorhynchos* | Resident | EN | 1988 | 1988 | 2002 | 35188.25 | 0.381491 |
| *Eulidia yarrellii* | Resident | CR | 1988 | 1988 | 2014 | 4704.3 | 3.377336 |
| *Pica asirensis* | Resident | EN | 2016 | 2016 | 2016 | 9173.31 | 3.27668 |
| *Thalurania watertonii* | Resident | EN | 1988 | 2004 | 2015 | 9527.11 | 4.41582 |
| *Setophaga chrysoparia* | Breeding | EN | 1988 | 1988 | 1994 | 29523.19 | 1.794759 |
| *Scytalopus novacapitalis* | Resident | EN | 1988 | 1988 | 2018 | 17238.56 | 3.795445 |
| *Padda oryzivora* | Resident | EN | 1988 | 1994 | 2018 | 58613.28 | 1.494832 |
| *Emberiza jankowskii* | Resident | EN | 1988 | 1988 | 2010 | 26122 | 3.761427 |
| *Nestor notabilis* | Resident | EN | 1988 | 1994 | 2017 | 58701.97 | 1.754967 |
| *Meiglyptes tristis* | Resident | EN | 2014 | 2014 | 2014 | 31229.31 | 4.534042 |
| *Calidris tenuirostris* | Breeding | EN | 1988 | 2010 | 2015 | 497651.8 | 0.311535 |
| *Zanda baudinii* | Resident | CR | 1988 | 1994 | 2022 | 39038.7 | 4.735737 |
| *Leucosticte australis* | nonbreeding | EN | 1988 | 2018 | 2018 | 63103.48 | 3.190949 |
| *Phylloscartes roquettei* | Resident | EN | 1988 | 1988 | 2000 | 128548.7 | 2.44939 |
| *Malacoptila minor* | Resident | EN | 2014 | 2014 | 2014 | 82181.24 | 4.511224 |
| *Ploceus megarhynchus* | Resident | EN | 1988 | 1988 | 2021 | 89656.65 | 4.61446 |
| *Sypheotides indicus* | Breeding | CR | 1988 | 1988 | 2021 | 245402.5 | 2.279915 |
| *Ardeotis nigriceps* | Resident | CR | 1988 | 1988 | 2011 | 226720.8 | 2.834336 |
| *Rynchops albicollis* | Breeding | EN | 1988 | 1994 | 2020 | 491281.5 | 1.862543 |
| *Haliaeetus leucoryphus* | Breeding | EN | 1988 | 1988 | 2017 | 251180.5 | 4.156656 |
| *Circus macrosceles* | Resident | EN | 2000 | 2000 | 2016 | 285997.9 | 4.686249 |
| *Rynchops albicollis* | nonbreeding | EN | 1988 | 1994 | 2020 | 514523 | 2.624767 |
| *Sypheotides indicus* | nonbreeding | CR | 1988 | 1988 | 2021 | 649064.8 | 2.174284 |
| *Sporophila palustris* | nonbreeding | EN | 1988 | 1988 | 1994 | 779998.7 | 2.715352 |
| *Vanellus gregarius* | Breeding | CR | 1988 | 1988 | 2004 | 583414.2 | 3.962432 |
| *Sterna acuticauda* | Resident | EN | 1988 | 1994 | 2012 | 1639707 | 2.532129 |
| *Numenius madagascariensis* | Breeding | EN | 1988 | 1994 | 2015 | 2385259 | 2.210434 |
| *Mergus squamatus* | nonbreeding | EN | 1988 | 1988 | 2002 | 1317436 | 4.057954 |
| *Gyps indicus* | Resident | CR | 2002 | 2002 | 2002 | 1107385 | 4.846996 |
| *Oroanassa magnifica* | Resident | EN | 1988 | 1988 | 1994 | 1315391 | 4.879383 |
| *Bubo blakistoni* | Resident | EN | 1988 | 1988 | 1994 | 1854362 | 4.508308 |


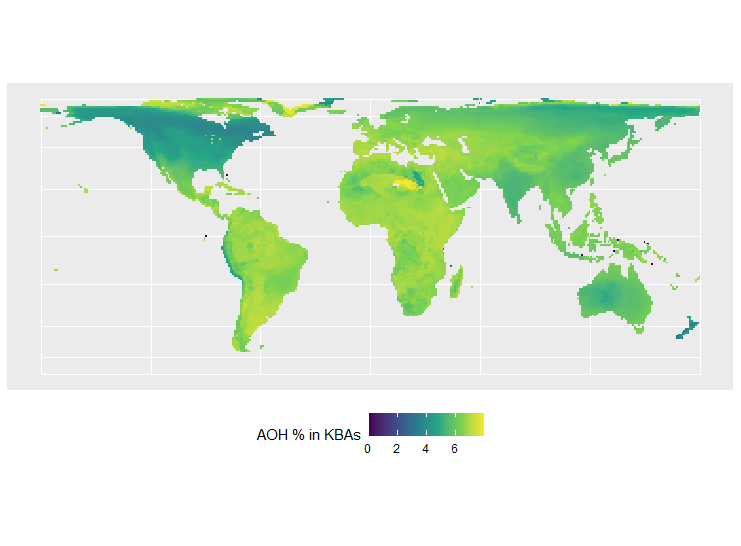


Appendix S6. The mean Area of Habitat (AOH) percentage of under-represented species (<8% of their AOH in KBAs) in KBAs per 100 km grid cell, (1874 species).


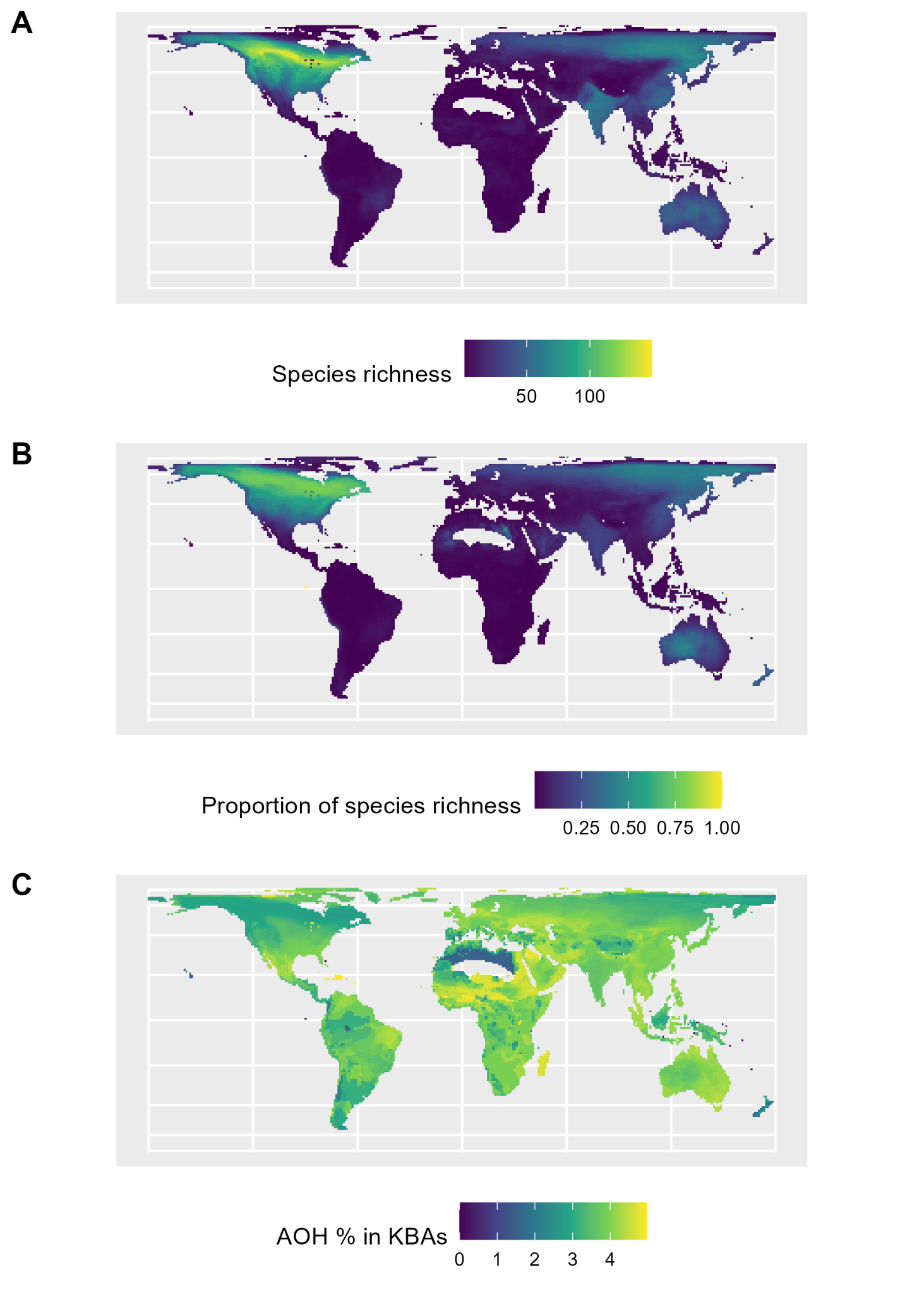


Appendix S7. (a) Species richness of very under-represented species by Key Biodiversity Areas (KBAs) (<5% of their suitable habitat overlaps with KBAs, 878 species shown); (b) the proportion of very under-represented species from the total data set of 10,969 range maps in each grid cell; (c) the mean Area of Habitat (AOH) percentage of very under-represented species in KBAs per 100 km grid cell, (878 species shown).

Appendix S8. The difference between the number of AOH maps with only one majorly important habitat (broad level 1 (L1) and fine level 2) under-represented by KBAs (the 2190 AOH maps with <8% of their suitable habitat overlapping KBAs) and 1000 random samples of 2190 AOH maps from the full set of 11,985 terrestrial avian AOH maps, as a percentage of the total number of AOH maps in that habitat; i.e. the difference between observed under-represented species by KBAs and randomly selected species, by habitat. A difference of 0 would mean the low overlap group is not different to the random samples, a negative difference would mean that category was less well represented than would be expected by chance, and a positive difference would mean that category was better represented than expected.

| Habitat | Number of AOH maps with under-representation | Total number of AOH maps with this habitat as its only majorly important one | Chance that the number of AOH maps being under-represented is random | Mean number of AOH maps selected by chance (binomial distribution) | Percentage of total number of AOH maps difference between random samples and observed under-represented AOH maps |
| --- | --- | --- | --- | --- | --- |
| Artificial/Aquatic & Marine L1 | 1 | 6 | 0.399755 | 1.600567 | 10.0094 |
| Artificial/Aquatic - Ponds (below 8ha) | 2 | 2 | 0.033384 | 1.10061 | -44.96951 |
| Artificial/Aquatic - Seasonally Flooded Agricultural Land | 1 | 4 | 0.398982 | 1.323894 | 8.09735 |
| Artificial/Terrestrial L1 | 6 | 38 | 0.161294 | 6.90991 | 2.3945 |
| Artificial/Terrestrial - Arable Land | 6 | 11 | 0.006268 | 2.230684 | -34.26651 |
| Artificial/Terrestrial - Pastureland | 3 | 5 | 0.040744 | 1.415873 | -31.68254 |
| Artificial/Terrestrial - Plantations | 1 | 2 | 0.298658 | 1.093656 | 4.68278 |
| Artificial/Terrestrial - Subtropical/Tropical Heavily Degraded Former Forest | 10 | 37 | 0.062202 | 6.767 | -8.737838 |
| Artificial/Terrestrial - Urban Areas | 1 | 5 | 0.407603 | 1.435312 | 8.70624 |
| Desert L1 | 4 | 12 | 0.10982 | 2.393443 | -13.38798 |
| Desert - Hot | 8 | 20 | 0.013897 | 3.696629 | -21.51685 |
| Desert - Temperate | 6 | 7 | 0.000213 | 1.732984 | -60.95737 |
| Forest L1 | 424 | 4598 | 7.13E-67 | 839.051 | 9.02677 |
| Forest - Boreal | 36 | 39 | 1.32E-23 | 6.981982 | -74.40517 |
| Forest - Subtropical/Tropical Dry | 20 | 222 | 4.93E-05 | 40.72 | 9.33333 |
| Forest - Subtropical/Tropical Mangrove Vegetation Above High Tide Level | 3 | 41 | 0.03043 | 7.625 | 11.2805 |
| Forest - Subtropical/Tropical Moist Lowland | 107 | 2152 | 1.50E-75 | 392.418 | 13.2629 |
| Forest - Subtropical/Tropical Moist Montane | 33 | 1433 | 1.04E-80 | 261.651 | 15.9561 |
| Forest - Subtropical/Tropical Swamp | 3 | 66 | 0.000842 | 12.261 | 14.0318 |
| Forest - Temperate | 104 | 240 | 2.19E-19 | 43.851 | -25.06208 |
| Grassland L1 | 78 | 253 | 4.93E-07 | 45.997 | -12.64941 |
| Grassland - Subtropical/Tropical Dry | 43 | 95 | 1.06E-09 | 17.359 | -26.99053 |
| Grassland - Subtropical/Tropical High Altitude | 4 | 66 | 0.002965 | 12.024 | 12.1576 |
| Grassland - Subtropical/Tropical Seasonally Wet/Flooded | 22 | 65 | 0.00119 | 11.872 | -15.58154 |
| Grassland - Temperate | 54 | 89 | 8.11E-19 | 16.247 | -42.4191 |
| Grassland - Tundra | 11 | 32 | 0.014126 | 5.836837 | -16.13488 |
| Marine Coastal/Supratidal L1 | 6 | 25 | 0.142545 | 4.563126 | -5.747495 |
| Marine Coastal/Supratidal - Coastal Brackish/Saline Lagoons/Marine Lakes | 3 | 8 | 0.124558 | 1.781638 | -15.22953 |
| Marine Coastal/Supratidal - Sea Cliffs and Rocky Offshore Islands | 3 | 8 | 0.124558 | 1.890547 | -13.86816 |
| Marine Intertidal L1 | 23 | 77 | 0.004732 | 13.875 | -11.85065 |
| Marine Intertidal - Mud Flats and Salt Flats | 2 | 13 | 0.282979 | 2.546739 | 4.20569 |
| Marine Intertidal - Rocky Shoreline | 1 | 1 | 0.182713 | 1 | 0 |
| Marine Intertidal - Salt Marshes (Emergent Grasses) | 2 | 6 | 0.223424 | 1.56447 | -7.258835 |
| Marine Neritic L1 | 23 | 76 | 0.00406 | 14.148 | -11.64737 |
| Marine Neritic - Estuaries | 4 | 7 | 0.021295 | 1.718421 | -32.59398 |
| Rocky areas (e.g. inland cliffs, mountain peaks) L1 | 9 | 42 | 0.129872 | 7.711 | -3.069048 |
| Savanna L1 | 9 | 60 | 0.113971 | 11.05 | 3.41667 |
| Savanna - Dry | 48 | 245 | 0.055699 | 44.716 | -1.340408 |
| Shrubland L1 | 80 | 452 | 0.046719 | 83.102 | 0.68628 |
| Shrubland - Boreal | 4 | 6 | 0.011167 | 1.562139 | -40.63102 |
| Shrubland - Mediterranean-type Shrubby Vegetation | 1 | 28 | 0.022031 | 5.164659 | 14.8738 |
| Shrubland - Subtropical/Tropical Dry | 72 | 235 | 1.59E-06 | 43.126 | -12.28681 |
| Shrubland - Subtropical/Tropical High Altitude | 21 | 129 | 0.079418 | 23.478 | 1.92093 |
| Shrubland - Subtropical/Tropical Moist | 16 | 76 | 0.092346 | 14.077 | -2.530263 |
| Shrubland - Temperate | 9 | 35 | 0.084434 | 6.418255 | -7.376415 |
| Wetlands (inland) L1 | 115 | 346 | 1.16E-11 | 63.443 | -14.90087 |
| Wetlands (inland) - Bogs, Marshes, Swamps, Fens, Peatlands | 55 | 179 | 1.99E-05 | 32.654 | -12.4838 |
| Wetlands (inland) - Permanent Freshwater Lakes (over 8ha) | 29 | 56 | 1.24E-08 | 10.345 | -33.3125 |
| Wetlands (inland) - Permanent Freshwater Marshes/Pools (under 8ha) | 3 | 11 | 0.20035 | 2.279461 | -6.550352 |
| Wetlands (inland) - Permanent Rivers/Streams/Creeks (includes waterfalls) | 27 | 74 | 0.000105 | 13.433 | -18.33378 |
| Wetlands (inland) - Permanent Saline, Brackish or Alkaline Lakes | 1 | 3 | 0.366134 | 1.222222 | 7.40741 |
| Wetlands (inland) - Permanent Saline, Brackish or Alkaline Marshes/Pools | 1 | 3 | 0.366134 | 1.189542 | 6.31808 |
| Wetlands (inland) - Seasonal/Intermittent Saline, Brackish or Alkaline Lakes and Flats | 1 | 1 | 0.182713 | 1 | 0 |
| Wetlands (inland) - Shrub Dominated Wetlands | 3 | 3 | 0.0061 | 1.196035 | -60.13216 |
| Wetlands (inland) - Tundra Wetlands (incl. pools and temporary waters from snowmelt) | 1 | 7 | 0.381167 | 1.686221 | 9.80316 |

Poorly represented Well represented


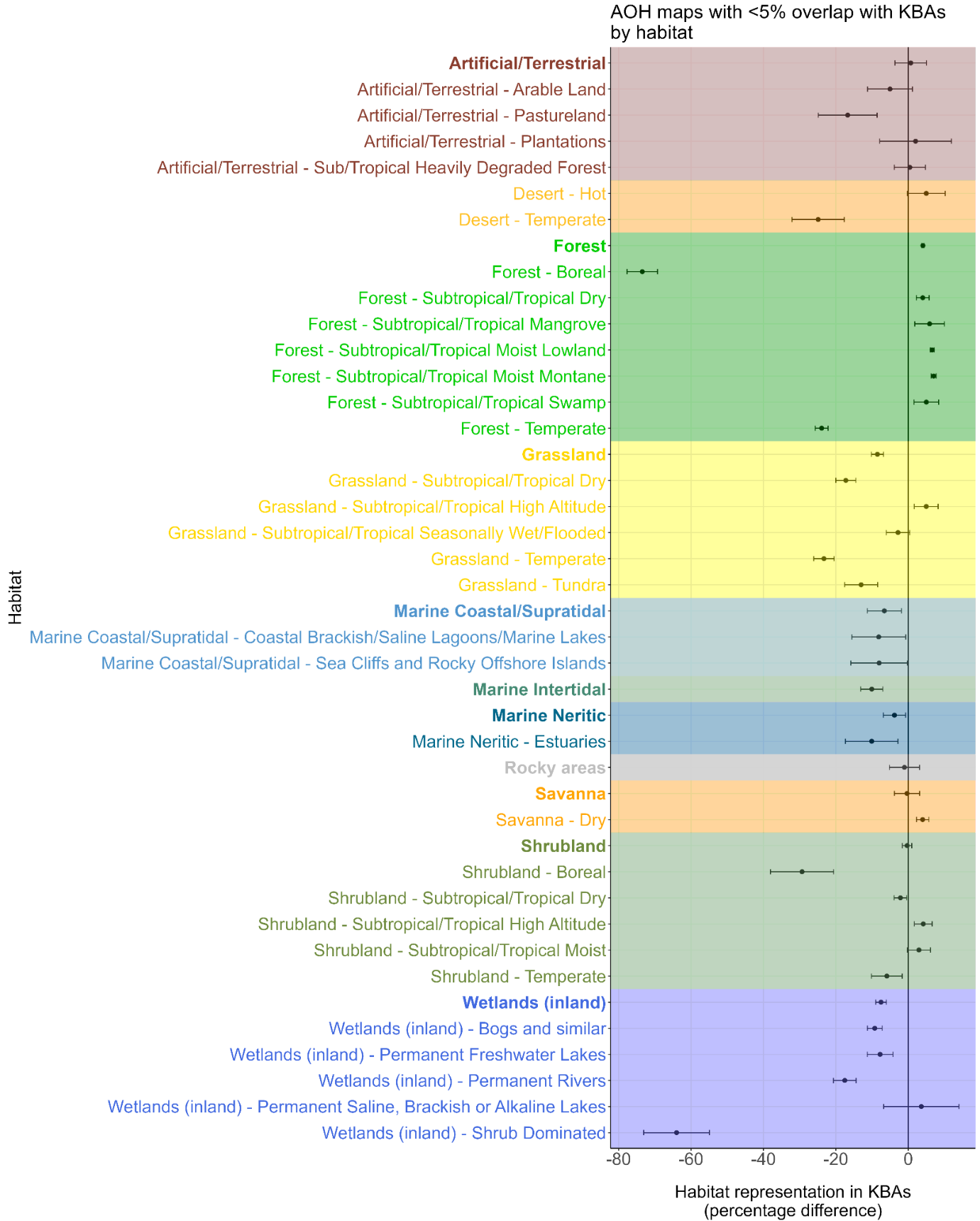


Appendix S9. The difference between the number of very under-represented AOH maps (the 970 AOH maps with <5% of their suitable habitat overlapping KBAs) with only one majorly important habitat (broad level 1 (L1) and fine level 2), and 1000 random samples of 970 AOH maps from the full set of 11,985 terrestrial avian AOH maps, as a percentage of the total number of AOH maps in that habitat; i.e. the difference between observed under-represented species by KBAs and randomly selected species, by habitat. A difference of 0 would mean the low overlap group is not different to the random samples, a negative difference would mean that category was represented less than expected by chance, and a positive difference would mean that category was better represented than expected.

Appendix S10. The difference between the number of AOH maps with only one majorly important habitat (broad level (in bold) and fine level) very under-represented by KBAs (the 970 AOH maps with <5% of their suitable habitat overlapping KBAs) and 1000 random samples of 970 AOH maps from the full set of 11,985 terrestrial avian AOH maps, as a percentage of the total number of AOH maps in that habitat; i.e. the difference between observed under-represented species by KBAs and randomly selected species, by habitat. A difference of 0 would mean the low overlap group is not different to the random samples, a negative difference would mean that category was less well represented than would be expected by chance, and a positive difference would mean that category was better represented than expected.

| Habitat | Number of AOH maps with under-representation | Total number of AOH maps with this habitat as its only majorly important one | Chance that the number of AOH maps being under-represented is random | Mean number of AOH maps selected by chance (binomial distribution) | Percentage of total number of AOH maps difference between random samples and observed under-represented AOH maps |
| --- | --- | --- | --- | --- | --- |
| Artificial/Terrestrial L1 | 3 | 38 | 0.233167 | 3.26569 | 0.699185 |
| Artificial/Terrestrial - Arable Land | 2 | 11 | 0.168541 | 1.447326 | -5.02431 |
| Artificial/Terrestrial - Pastureland | 2 | 5 | 0.050844 | 1.163842 | -16.7232 |
| Artificial/Terrestrial - Plantations | 1 | 2 | 0.148757 | 1.040698 | 2.034884 |
| Artificial/Terrestrial - Subtropical/Tropical Heavily Degraded Former Forest | 3 | 37 | 0.233669 | 3.161826 | 0.437367 |
| Desert - Hot | 1 | 20 | 0.325665 | 1.995128 | 4.975639 |
| Desert - Temperate | 3 | 7 | 0.013236 | 1.257919 | -24.8869 |
| Forest L1 | 186 | 4598 | 8.14E-29 | 372.195 | 4.049478 |
| Forest - Boreal | 32 | 39 | 9.76E-29 | 3.346749 | -73.4699 |
| Forest - Subtropical/Tropical Dry | 9 | 222 | 0.007119 | 17.905 | 4.011261 |
| Forest - Subtropical/Tropical Mangrove Vegetation Above High Tide Level | 1 | 41 | 0.113466 | 3.418724 | 5.899328 |
| Forest - Subtropical/Tropical Moist Lowland | 31 | 2152 | 5.30E-43 | 174.066 | 6.648048 |
| Forest - Subtropical/Tropical Moist Montane | 15 | 1433 | 7.02E-34 | 115.523 | 7.014864 |
| Forest - Subtropical/Tropical Swamp | 2 | 66 | 0.063387 | 5.294472 | 4.991625 |
| Forest - Temperate | 77 | 240 | 1.24E-26 | 19.603 | -23.9154 |
| Grassland L1 | 42 | 253 | 4.21E-06 | 20.464 | -8.51225 |
| Grassland - Subtropical/Tropical Dry | 24 | 95 | 3.05E-07 | 7.614615 | -17.2478 |
| Grassland - Subtropical/Tropical High Altitude | 2 | 66 | 0.063387 | 5.283838 | 4.975513 |
| Grassland - Subtropical/Tropical Seasonally Wet/Flooded | 7 | 65 | 0.118492 | 5.162325 | -2.82719 |
| Grassland - Temperate | 28 | 89 | 1.66E-10 | 7.279279 | -23.2817 |
| Grassland - Tundra | 7 | 32 | 0.009279 | 2.833876 | -13.0191 |
| Marine Coastal/Supratidal L1 | 4 | 25 | 0.09222 | 2.349261 | -6.60296 |
| Marine Coastal/Supratidal - Coastal Brackish/Saline Lagoons/Marine Lakes | 2 | 8 | 0.110523 | 1.348361 | -8.14549 |
| Marine Coastal/Supratidal - Sea Cliffs and Rocky Offshore Islands | 2 | 8 | 0.110523 | 1.356557 | -8.04303 |
| Marine Intertidal L1 | 14 | 77 | 0.002131 | 6.24024 | -10.0776 |
| Marine Neritic L1 | 9 | 76 | 0.074305 | 6.107107 | -3.80644 |
| Marine Neritic - Estuaries | 2 | 7 | 0.090191 | 1.293333 | -10.0952 |
| Rocky areas (eg. inland cliffs, mountain peaks) L1 | 4 | 42 | 0.194367 | 3.561602 | -1.04381 |
| Savanna L1 | 5 | 60 | 0.182823 | 4.795363 | -0.34106 |
| Savanna - Dry | 10 | 245 | 0.005239 | 19.74 | 3.97551 |
| Shrubland L1 | 38 | 452 | 0.0655 | 36.559 | -0.31881 |
| Shrubland - Boreal | 3 | 6 | 0.008229 | 1.240506 | -29.3249 |
| Shrubland - Subtropical/Tropical Dry | 24 | 235 | 0.044266 | 18.94 | -2.15319 |
| Shrubland - Subtropical/Tropical High Altitude | 5 | 129 | 0.027261 | 10.365 | 4.158915 |
| Shrubland - Subtropical/Tropical Moist | 4 | 76 | 0.12641 | 6.247495 | 2.95723 |
| Shrubland - Temperate | 5 | 35 | 0.089612 | 2.932131 | -5.9082 |
| Wetlands (inland) L1 | 54 | 346 | 1.53E-06 | 27.988 | -7.51792 |
| Wetlands (inland) - Bogs, Marshes, Swamps, Fens, Peatlands | 31 | 179 | 2.83E-05 | 14.442 | -9.25028 |
| Wetlands (inland) - Permanent Freshwater Lakes (over 8ha) | 9 | 56 | 0.021367 | 4.643794 | -7.77894 |
| Wetlands (inland) - Permanent Rivers/Streams/Creeks (includes waterfalls) | 19 | 74 | 3.71E-06 | 6.023023 | -17.5365 |
| Wetlands (inland) - Permanent Saline, Brackish or Alkaline Lakes | 1 | 3 | 0.205078 | 1.108374 | 3.612479 |
| Wetlands (inland) - Shrub Dominated Wetlands | 3 | 3 | 0.00053 | 1.080169 | -63.9944 |

Appendix S11. The difference between the number of AOH maps in each order under-represented by KBAs (the 2190 AOH maps with <8% of their suitable habitat overlapping KBAs) and 1000 random samples of 2190 AOH maps from the full set of 11,985 terrestrial avian AOH maps, as a percentage of the total number of AOH maps in that order; i.e. the difference between observed under-represented species by KBAs and randomly selected species, by order. A difference of 0 would mean the low overlap group is not different to the random samples, a negative difference would mean that category was less well represented than would be expected by chance, and a positive difference would mean that category was better represented than expected.

| Order | Number of AOH maps with under- representation | Total number of AOH maps | Chance that the number of AOH maps being under-represented is random | Mean number of AOH maps selected by chance (binomial distribution) | Percentage of total number of AOH maps difference between random samples and observed under-represented AOH maps |
| --- | --- | --- | --- | --- | --- |
| Accipitriformes | 68 | 320 | 0.021834 | 58.521 | -2.96219 |
| Anseriformes | 116 | 246 | 3.64e-25 | 44.669 | -28.9963 |
| Bucerotiformes | 6 | 72 | 0.009575 | 13.176 | 9.966667 |
| Caprimulgiformes | 74 | 639 | 1.10e-06 | 117.504 | 6.808138 |
| Charadriiformes | 164 | 454 | 1.21e-19 | 82.699 | -17.9077 |
| Ciconiiformes | 7 | 29 | 0.125305 | 5.265531 | -5.98093 |
| Coliiformes | 1 | 6 | 0.399755 | 1.541547 | 9.025788 |
| Columbiformes | 50 | 349 | 0.008803 | 63.839 | 3.96533 |
| Coraciiformes | 17 | 203 | 3.38e-05 | 36.915 | 9.810345 |
| Cuculiformes | 16 | 188 | 7.90e-05 | 34.193 | 9.677128 |
| Falconiformes | 20 | 77 | 0.025633 | 14.038 | -7.74286 |
| Galliformes | 66 | 309 | 0.021453 | 56.399 | -3.10712 |
| Gaviiformes | 2 | 10 | 0.299053 | 2.1 | 1 |
| Gruiformes | 56 | 201 | 0.000249 | 36.701 | -9.60149 |
| Musophagiformes | 4 | 24 | 0.209382 | 4.308853 | 1.286888 |
| Otidiformes | 12 | 31 | 0.004226 | 5.721443 | -20.2534 |
| Passeriformes | 1257 | 7288 | 0.000922 | 1331.56 | 1.023052 |
| Pelecaniformes | 38 | 155 | 0.011429 | 28.449 | -6.16194 |
| Phoenicopteriformes | 1 | 9 | 0.327347 | 2.058057 | 11.75619 |
| Piciformes | 65 | 492 | 0.000547 | 89.575 | 4.994919 |
| Podicipediformes | 18 | 31 | 7.71e-07 | 5.711423 | -39.6406 |
| Psittaciformes | 46 | 402 | 5.47e-05 | 73.629 | 6.872886 |
| Pterocliformes | 7 | 20 | 0.038254 | 3.709054 | -16.4547 |
| Strigiformes | 44 | 243 | 0.066183 | 44.518 | 0.213169 |
| Struthioniformes | 19 | 57 | 0.002805 | 10.306 | -15.2526 |
| Suliformes | 1 | 23 | 0.049629 | 4.281818 | 14.26877 |
| Trogoniformes | 1 | 43 | 0.00164 | 7.923 | 16.1 |


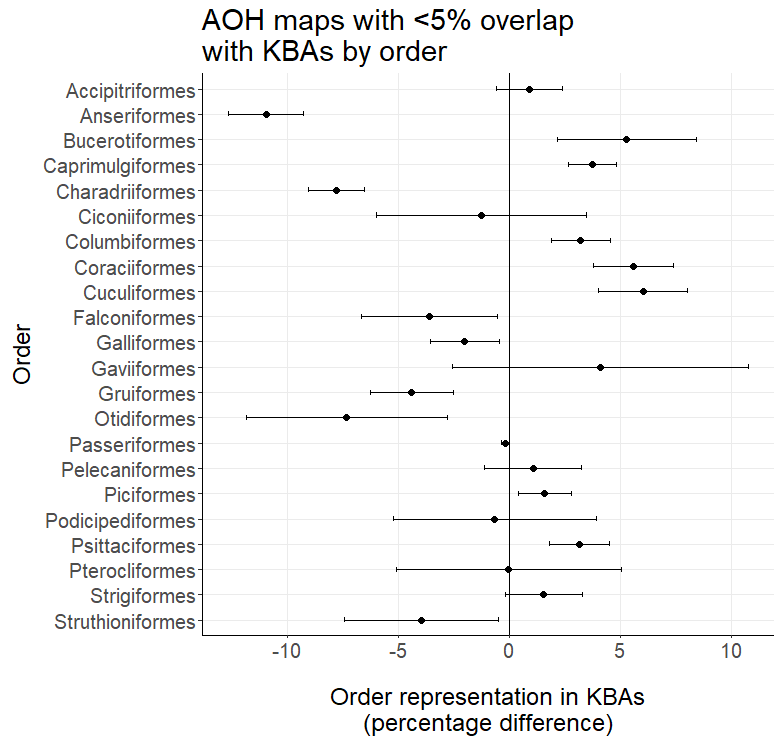


Poorly represented Well represented

Appendix S12. The difference between the number of AOH maps from each order in the <5% overlap group (the 970 AOH maps with <5% of their suitable habitat overlapping KBAs) and 1000 samples of 970 AOH maps from the full set of 11,985 terrestrial avian AOH maps, as a percentage of the total number of AOH maps in that order. A difference of 0 would mean the low overlap group is not different to the random samples, a negative difference would mean that category was less well represented than would be randomly expected, and a positive difference would mean that category was better represented than randomly expected.

Appendix S13. The difference between the number of AOH maps in each order very under-represented by KBAs (the 970 AOH maps with <5% of their suitable habitat overlapping KBAs) and 1000 random samples of 970 AOH maps from the full set of 11,985 terrestrial avian AOH maps, as a percentage of the total number of AOH maps in that order; i.e. the difference between observed under-represented species by KBAs and randomly selected species, by order. A difference of 0 would mean the low overlap group is not different to the random samples, a negative difference would mean that category was less well represented than would be expected by chance, and a positive difference would mean that category was better represented than expected.

| Order | Number of AOH maps with under- representation | Total number of AOH maps | Chance that the number of AOH maps being under-represented is random | Mean number of AOH maps selected by chance (binomial distribution) | Percentage of total number of AOH maps difference between random samples and observed under-represented AOH maps |
| --- | --- | --- | --- | --- | --- |
| Accipitriformes | 23 | 320 | 0.07167 | 25.875 | 0.898438 |
| Anseriformes | 47 | 246 | 2.03E-08 | 19.978 | -10.9846 |
| Bucerotiformes | 2 | 72 | 0.045523 | 5.798597 | 5.275829 |
| Caprimulgiformes | 28 | 639 | 6.96E-05 | 51.882 | 3.737402 |
| Charadriiformes | 72 | 454 | 2.08E-08 | 36.577 | -7.80242 |
| Ciconiiformes | 3 | 29 | 0.21585 | 2.630482 | -1.2742 |
| Columbiformes | 17 | 349 | 0.005953 | 28.187 | 3.205444 |
| Coraciiformes | 5 | 203 | 0.000525 | 16.316 | 5.574384 |
| Cuculiformes | 4 | 188 | 0.00039 | 15.31 | 6.015957 |
| Falconiformes | 9 | 77 | 0.07733 | 6.205 | -3.62987 |
| Galliformes | 31 | 309 | 0.036286 | 24.747 | -2.02362 |
| Gaviiformes | 1 | 10 | 0.378656 | 1.410072 | 4.100719 |
| Gruiformes | 25 | 201 | 0.009225 | 16.136 | -4.40995 |
| Otidiformes | 5 | 31 | 0.065735 | 2.722401 | -7.34709 |
| Passeriformes | 604 | 7288 | 0.014091 | 589.729 | -0.19582 |
| Pelecaniformes | 11 | 155 | 0.111227 | 12.63 | 1.051613 |
| Piciformes | 32 | 492 | 0.029867 | 39.735 | 1.572154 |
| Podicipediformes | 3 | 31 | 0.224291 | 2.792735 | -0.6686 |
| Psittaciformes | 20 | 402 | 0.00448 | 32.567 | 3.126119 |
| Pterocliformes | 2 | 20 | 0.272422 | 1.992718 | -0.03641 |
| Strigiformes | 16 | 243 | 0.069157 | 19.724 | 1.53251 |
| Struthioniformes | 7 | 57 | 0.08839 | 4.739879 | -3.96513 |

Poorly represented Well represented


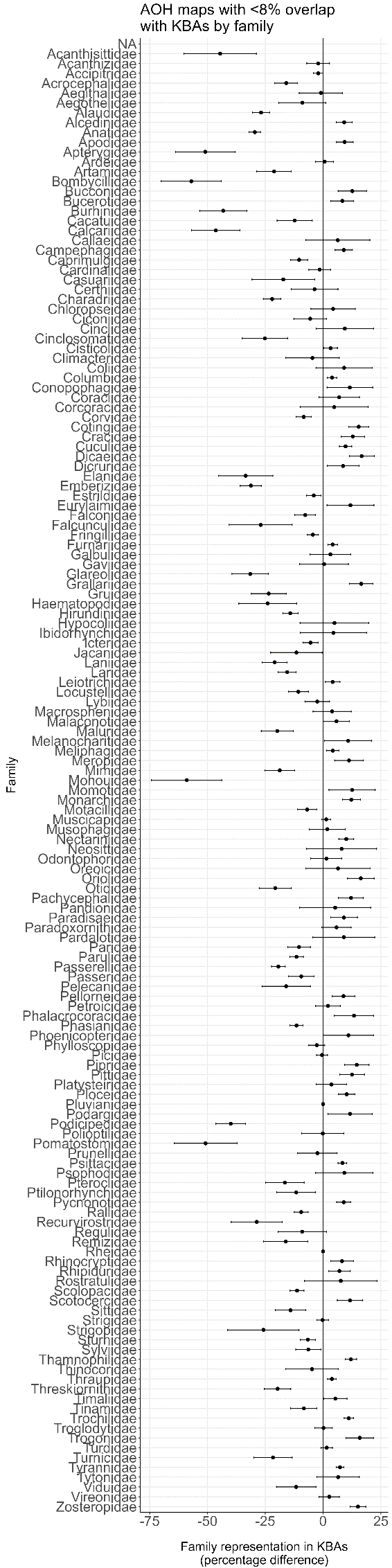


Appendix S14. The difference between the number of AOH maps from families in the <8% overlap group (the 2190 AOH maps with <8% of their suitable habitat overlapping KBAs) and 1000 random samples of 2190 AOH maps from the full set of 11,985 terrestrial avian AOH maps, as a percentage of the total number of AOH maps in that family. A difference of 0 would mean the low overlap group is not different to the random samples, a negative difference would mean that category was less well represented than would be randomly expected, and a positive difference would mean that category was better represented than randomly expected.

Appendix S15. The difference between the number of AOH maps in each family under-represented by KBAs (the 2190 AOH maps with <8% of their suitable habitat overlapping KBAs) and 1000 random samples of 2190 AOH maps from the full set of 11,985 terrestrial avian AOH maps, as a percentage of the total number of AOH maps in that family; i.e. the difference between observed under-represented species by KBAs and randomly selected species, by family. A difference of 0 would mean the low overlap group is not different to the random samples, a negative difference would mean that category was less well represented than would be expected by chance, and a positive difference would mean that category was better represented than expected.

| Family | Number of AOH maps with under- representation | Total number of AOH maps | Chance that the number of AOH maps being under-represented is random | Mean number of AOH maps selected by chance (binomial distribution) | Percentage of total number of AOH maps difference between random samples and observed under-represented AOH maps |
| --- | --- | --- | --- | --- | --- |
| Acanthisittidae | 2 | 2 | 0.033384 | 1.109677 | -44.5161 |
| Acanthizidae | 13 | 64 | 0.112875 | 11.65 | -2.10938 |
| Accipitridae | 63 | 310 | 0.03677 | 56.555 | -2.07903 |
| Acrocephalidae | 21 | 61 | 0.001196 | 11.271 | -15.9492 |
| Aegithalidae | 3 | 15 | 0.246499 | 2.857445 | -0.95037 |
| Aegothelidae | 3 | 10 | 0.178284 | 2.104651 | -8.95349 |
| Alaudidae | 47 | 104 | 2.00E-10 | 19.087 | -26.8394 |
| Alcedinidae | 11 | 121 | 0.002222 | 22.052 | 9.133884 |
| Anatidae | 116 | 242 | 6.22E-26 | 44.595 | -29.5062 |
| Apodidae | 10 | 112 | 0.002708 | 20.456 | 9.335714 |
| Apterygidae | 4 | 5 | 0.004554 | 1.452713 | -50.9457 |
| Ardeidae | 17 | 97 | 0.104086 | 17.664 | 0.684536 |
| Artamidae | 10 | 25 | 0.006572 | 4.700201 | -21.1992 |
| Bombycillidae | 5 | 6 | 0.000999 | 1.58156 | -56.974 |
| Bucconidae | 2 | 38 | 0.016443 | 6.810811 | 12.66003 |
| Bucerotidae | 6 | 61 | 0.031312 | 11.08 | 8.327869 |
| Burhinidae | 7 | 11 | 0.001001 | 2.248588 | -43.1947 |
| Cacatuidae | 7 | 23 | 0.066043 | 4.175703 | -12.2796 |
| Calcariidae | 8 | 12 | 0.000274 | 2.431072 | -46.4077 |
| Callaeidae | 1 | 3 | 0.366134 | 1.190805 | 6.360153 |
| Campephagidae | 10 | 107 | 0.004611 | 19.576 | 8.949533 |
| Caprimulgidae | 33 | 115 | 0.002012 | 21.11 | -10.3391 |
| Cardinalidae | 13 | 66 | 0.117362 | 12.026 | -1.47576 |
| Casuariidae | 2 | 4 | 0.133795 | 1.312057 | -17.1986 |
| Certhiidae | 3 | 13 | 0.231964 | 2.524625 | -3.65673 |
| Charadriidae | 40 | 99 | 1.65E-07 | 18.2 | -22.0202 |
| Chloropseidae | 2 | 13 | 0.282979 | 2.565124 | 4.347106 |
| Ciconiidae | 7 | 29 | 0.125305 | 5.38995 | -5.5519 |
| Cinclidae | 1 | 6 | 0.399755 | 1.563845 | 9.397418 |
| Cinclosomatidae | 5 | 11 | 0.028037 | 2.232584 | -25.1583 |
| Cisticolidae | 24 | 161 | 0.046267 | 29.262 | 3.268323 |
| Climacteridae | 2 | 7 | 0.255642 | 1.677835 | -4.60236 |
| Coliidae | 1 | 6 | 0.399755 | 1.546875 | 9.114583 |
| Columbidae | 50 | 349 | 0.008803 | 63.497 | 3.867335 |
| Conopophagidae | 1 | 12 | 0.238271 | 2.389254 | 11.57712 |
| Coraciidae | 2 | 17 | 0.220139 | 3.179834 | 6.940198 |
| Corcoracidae | 1 | 2 | 0.298658 | 1.096096 | 4.804805 |
| Corvidae | 36 | 136 | 0.004831 | 24.629 | -8.36103 |
| Cotingidae | 2 | 73 | 5.27E-05 | 13.251 | 15.41233 |
| Cracidae | 3 | 55 | 0.004443 | 10.075 | 12.86364 |
| Cuculidae | 16 | 188 | 7.90E-05 | 34.3 | 9.734043 |
| Dicaeidae | 1 | 55 | 0.000186 | 10.188 | 16.70545 |
| Dicruridae | 3 | 30 | 0.106647 | 5.591592 | 8.638639 |
| Elanidae | 4 | 7 | 0.021295 | 1.658278 | -33.4532 |
| Emberizidae | 35 | 71 | 2.25E-09 | 12.828 | -31.2282 |
| Estrildidae | 31 | 140 | 0.041519 | 25.375 | -4.01786 |
| Eurylaimidae | 1 | 12 | 0.238271 | 2.421053 | 11.84211 |
| Falconidae | 20 | 77 | 0.025633 | 14.057 | -7.71818 |
| Falcunculidae | 2 | 3 | 0.081853 | 1.190578 | -26.9807 |
| Fringillidae | 57 | 252 | 0.01328 | 45.918 | -4.39762 |
| Furnariidae | 47 | 332 | 0.008425 | 60.728 | 4.13494 |
| Galbulidae | 3 | 19 | 0.234221 | 3.607287 | 3.19625 |
| Gaviidae | 2 | 10 | 0.299053 | 2.042973 | 0.429733 |
| Glareolidae | 12 | 24 | 0.000332 | 4.441978 | -31.4918 |
| Grallariidae | 1 | 55 | 0.000186 | 10.081 | 16.51091 |
| Gruidae | 10 | 24 | 0.004825 | 4.356636 | -23.514 |
| Haematopodidae | 3 | 6 | 0.066598 | 1.562849 | -23.9525 |
| Hirundinidae | 42 | 130 | 4.76E-05 | 23.575 | -14.1731 |
| Hypocoliidae | 1 | 2 | 0.298658 | 1.098507 | 4.925373 |
| Ibidorhynchidae | 1 | 2 | 0.298658 | 1.089595 | 4.479769 |
| Icteridae | 32 | 135 | 0.023179 | 24.64 | -5.45185 |
| Jacanidae | 3 | 9 | 0.152699 | 1.962696 | -11.5256 |
| Laniidae | 20 | 51 | 0.000256 | 9.321 | -20.9392 |
| Laridae | 35 | 103 | 6.17E-05 | 18.988 | -15.5456 |
| Leiotrichidae | 20 | 140 | 0.043444 | 25.815 | 4.153571 |
| Locustellidae | 23 | 79 | 0.006323 | 14.556 | -10.6886 |
| Lybiidae | 11 | 53 | 0.120583 | 9.664 | -2.52075 |
| Macrosphenidae | 3 | 21 | 0.214735 | 3.821138 | 3.910182 |
| Malaconotidae | 6 | 48 | 0.095334 | 8.782 | 5.795833 |
| Maluridae | 11 | 29 | 0.006938 | 5.260521 | -19.7913 |
| Melanocharitidae | 1 | 11 | 0.267244 | 2.192785 | 10.8435 |
| Meliphagidae | 27 | 192 | 0.024463 | 35.107 | 4.222396 |
| Meropidae | 3 | 41 | 0.03043 | 7.602 | 11.22439 |
| Mimidae | 14 | 38 | 0.003525 | 6.882 | -18.7316 |
| Mohouidae | 3 | 3 | 0.0061 | 1.230937 | -58.9688 |
| Momotidae | 1 | 14 | 0.185681 | 2.759219 | 12.56585 |
| Monarchidae | 6 | 101 | 0.000223 | 18.334 | 12.21188 |
| Motacillidae | 22 | 86 | 0.024045 | 16.065 | -6.90116 |
| Muscicapidae | 70 | 412 | 0.041504 | 75.649 | 1.371117 |
| Musophagidae | 4 | 24 | 0.209382 | 4.443662 | 1.848592 |
| Nectariniidae | 12 | 146 | 0.000309 | 26.645 | 10.03082 |
| Neosittidae | 1 | 3 | 0.366134 | 1.241535 | 8.051166 |
| Odontophoridae | 6 | 35 | 0.173716 | 6.503511 | 1.438602 |
| Oreoicidae | 1 | 3 | 0.366134 | 1.194131 | 6.471031 |
| Oriolidae | 1 | 45 | 0.001147 | 8.344 | 16.32 |
| Otididae | 12 | 31 | 0.004226 | 5.588176 | -20.6833 |
| Pachycephalidae | 3 | 50 | 0.009103 | 9.038 | 12.076 |
| Pandionidae | 1 | 2 | 0.298658 | 1.104615 | 5.230769 |
| Paradisaeidae | 4 | 43 | 0.052606 | 7.884 | 9.032558 |
| Paradoxornithidae | 5 | 39 | 0.122976 | 7.271271 | 5.823772 |
| Pardalotidae | 1 | 5 | 0.407603 | 1.448947 | 8.97893 |
| Paridae | 18 | 63 | 0.015198 | 11.444 | -10.4063 |
| Parulidae | 51 | 171 | 8.18E-05 | 31.382 | -11.4725 |
| Passerellidae | 65 | 172 | 9.19E-10 | 31.756 | -19.3279 |
| Passeridae | 13 | 47 | 0.037322 | 8.569 | -9.42766 |
| Pelecanidae | 4 | 11 | 0.089581 | 2.241881 | -15.9829 |
| Pellorneidae | 6 | 63 | 0.025593 | 11.563 | 8.830159 |
| Petroicidae | 9 | 55 | 0.134432 | 10.161 | 2.110909 |
| Phalacrocoracidae | 1 | 19 | 0.091889 | 3.544625 | 13.39276 |
| Phasianidae | 57 | 191 | 3.46E-05 | 35.079 | -11.477 |
| Phoenicopteridae | 1 | 9 | 0.327347 | 1.985899 | 10.95443 |
| Phylloscopidae | 27 | 128 | 0.06201 | 23.517 | -2.72109 |
| Picidae | 49 | 261 | 0.061755 | 47.832 | -0.44751 |
| Pipridae | 2 | 53 | 0.001563 | 9.727 | 14.57925 |
| Pittidae | 3 | 53 | 0.005939 | 9.642 | 12.53208 |
| Platysteiridae | 5 | 34 | 0.162988 | 6.235 | 3.632353 |
| Ploceidae | 10 | 121 | 0.000985 | 22.378 | 10.22975 |
| Pluvianidae | 1 | 1 | 0.182713 | 1 | 0 |
| Podargidae | 1 | 13 | 0.210963 | 2.516447 | 11.66498 |
| Podicipedidae | 18 | 31 | 7.71E-07 | 5.633267 | -39.8927 |
| Polioptilidae | 3 | 16 | 0.247951 | 2.980208 | -0.1237 |
| Pomatostomidae | 4 | 5 | 0.004554 | 1.459375 | -50.8125 |
| Prunellidae | 4 | 19 | 0.20945 | 3.529949 | -2.47395 |
| Psittacidae | 37 | 376 | 2.13E-06 | 68.473 | 8.370479 |
| Psophodidae | 1 | 5 | 0.407603 | 1.460912 | 9.218241 |
| Pteroclidae | 7 | 20 | 0.038254 | 3.700611 | -16.4969 |
| Ptilonorhynchidae | 6 | 20 | 0.085555 | 3.677618 | -11.6119 |
| Pycnonotidae | 15 | 159 | 0.000826 | 29.174 | 8.914465 |
| Rallidae | 46 | 166 | 0.000822 | 30.309 | -9.45241 |
| Recurvirostridae | 5 | 10 | 0.018712 | 2.132875 | -28.6712 |
| Regulidae | 3 | 10 | 0.178284 | 2.098851 | -9.01149 |
| Remizidae | 5 | 14 | 0.066326 | 2.740149 | -16.1418 |
| Rheidae | 1 | 1 | 0.182713 | 1 | 0 |
| Rhinocryptidae | 6 | 60 | 0.034544 | 10.926 | 8.21 |
| Rhipiduridae | 7 | 63 | 0.046591 | 11.453 | 7.068254 |
| Rostratulidae | 1 | 3 | 0.366134 | 1.229614 | 7.653791 |
| Scolopacidae | 46 | 156 | 0.000216 | 28.455 | -11.2468 |
| Scotocercidae | 3 | 47 | 0.013794 | 8.482 | 11.66383 |
| Sittidae | 11 | 34 | 0.020922 | 6.22 | -14.0588 |
| Strigidae | 42 | 227 | 0.067774 | 41.594 | -0.17885 |
| Strigopidae | 2 | 3 | 0.081853 | 1.226293 | -25.7902 |
| Sturnidae | 32 | 129 | 0.014805 | 23.513 | -6.57907 |
| Sylviidae | 12 | 49 | 0.073135 | 8.884 | -6.35918 |
| Thamnophilidae | 15 | 242 | 3.05E-08 | 44.133 | 12.03843 |
| Thinocoridae | 2 | 7 | 0.255642 | 1.664474 | -4.79323 |
| Thraupidae | 61 | 423 | 0.005934 | 77.058 | 3.796217 |
| Threskiornithidae | 17 | 45 | 0.001094 | 8.154 | -19.6578 |
| Timaliidae | 7 | 54 | 0.091666 | 9.877 | 5.327778 |
| Tinamidae | 12 | 45 | 0.051096 | 8.259 | -8.31333 |
| Trochilidae | 27 | 377 | 3.23E-10 | 68.868 | 11.10557 |
| Troglodytidae | 18 | 101 | 0.102523 | 18.189 | 0.187129 |
| Trogonidae | 1 | 43 | 0.00164 | 7.815 | 15.84884 |
| Turdidae | 38 | 227 | 0.058974 | 41.596 | 1.584141 |
| Turnicidae | 8 | 20 | 0.013897 | 3.673096 | -21.6345 |
| Tyrannidae | 58 | 530 | 1.13E-06 | 96.763 | 7.313774 |
| Tytonidae | 2 | 16 | 0.237664 | 3.043022 | 6.518888 |
| Viduidae | 6 | 20 | 0.085555 | 3.674562 | -11.6272 |
| Vireonidae | 12 | 77 | 0.102525 | 14.055 | 2.668831 |
| Zosteropidae | 4 | 122 | 4.48E-07 | 22.431 | 15.10738 |


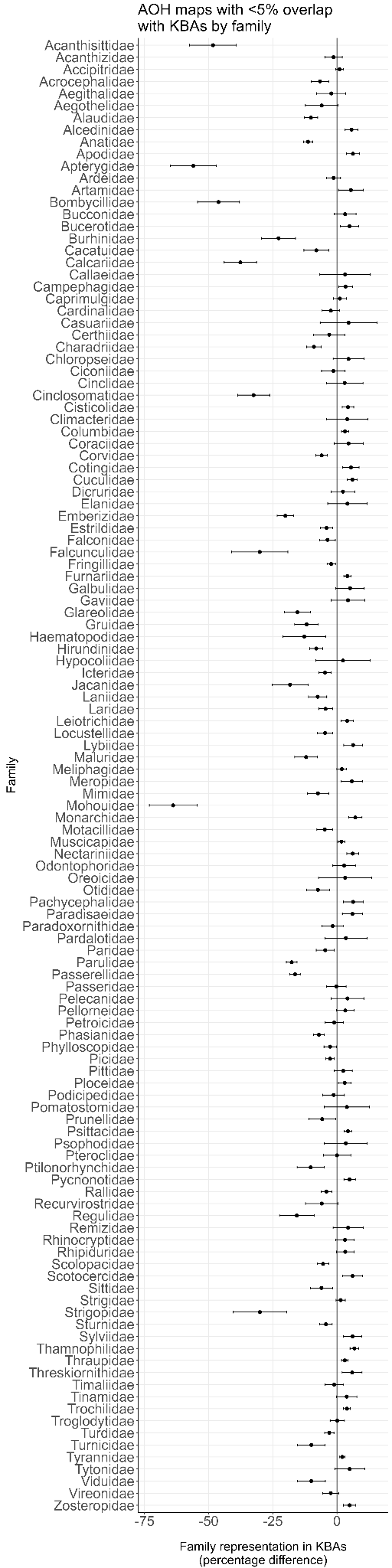

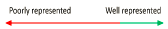


Appendix S16. The difference between the number of AOH maps from families very under-represented by KBAs (the 970 AOH maps with <5% of their suitable habitat overlapping KBAs) and 1000 samples of 970 AOH maps from the full set of 11,985 terrestrial avian AOH maps, as a percentage of the total number of AOH maps in that family. A difference of 0 would mean the low overlap group is not different to the random samples, a negative difference would mean that category was less well represented than would be randomly expected, and a positive difference would mean that category was better represented than randomly expected.

Appendix S17. The difference between the number of AOH maps in each family very under-represented by KBAs (the 970 AOH maps with <5% of their suitable habitat overlapping KBAs) and 1000 random samples of 970 AOH maps from the full set of 11,985 terrestrial avian AOH maps, as a percentage of the total number of AOH maps in that family; i.e. the difference between observed under-represented species by KBAs and randomly selected species, by family. A difference of 0 would mean the low overlap group is not different to the random samples, a negative difference would mean that category was less well represented than would be expected by chance, and a positive difference would mean that category was better represented than expected.

| Family | Number of AOH maps with under- representation | Total number of AOH maps | Chance that the number of AOH maps being under-represented is random | Mean number of AOH maps selected by chance (binomial distribution) | Percentage of total number of AOH maps difference between random samples and observed under-represented AOH maps |
| --- | --- | --- | --- | --- | --- |
| Acanthisittidae | 2 | 2 | 0.006549 | 1.034286 | -48.2857 |
| Acanthizidae | 6 | 64 | 0.15768 | 5.152457 | -1.32429 |
| Accipitridae | 22 | 310 | 0.070903 | 25.201 | 1.032581 |
| Acrocephalidae | 9 | 61 | 0.032074 | 4.977889 | -6.59362 |
| Aegithalidae | 2 | 15 | 0.229578 | 1.668508 | -2.20994 |
| Aegothelidae | 2 | 10 | 0.150039 | 1.407534 | -5.92466 |
| Alaudidae | 19 | 104 | 0.000413 | 8.461 | -10.1337 |
| Alcedinidae | 3 | 121 | 0.007226 | 9.883 | 5.68843 |
| Anatidae | 47 | 242 | 1.21E-08 | 19.797 | -11.2409 |
| Apodidae | 2 | 112 | 0.003786 | 9.009009 | 6.258044 |
| Apterygidae | 4 | 5 | 0.000197 | 1.202778 | -55.9444 |
| Ardeidae | 9 | 97 | 0.126684 | 7.776777 | -1.26105 |
| Artamidae | 1 | 25 | 0.26695 | 2.368119 | 5.472477 |
| Bombycillidae | 4 | 6 | 0.000543 | 1.230971 | -46.1505 |
| Bucconidae | 2 | 38 | 0.220667 | 3.221184 | 3.213642 |
| Bucerotidae | 2 | 61 | 0.082466 | 5.016113 | 4.944447 |
| Burhinidae | 4 | 11 | 0.007841 | 1.497521 | -22.7498 |
| Cacatuidae | 4 | 23 | 0.076423 | 2.163991 | -7.98265 |
| Calcariidae | 6 | 12 | 0.000156 | 1.483516 | -37.6374 |
| Callaeidae | 1 | 3 | 0.205078 | 1.096491 | 3.216374 |
| Campephagidae | 5 | 107 | 0.067409 | 8.624 | 3.386916 |
| Caprimulgidae | 8 | 115 | 0.130358 | 9.317 | 1.145217 |
| Cardinalidae | 7 | 66 | 0.121824 | 5.439759 | -2.364 |
| Casuariidae | 1 | 4 | 0.251308 | 1.180602 | 4.51505 |
| Certhiidae | 2 | 13 | 0.2019 | 1.611354 | -2.98959 |
| Charadriidae | 17 | 99 | 0.001494 | 8.129 | -8.96061 |
| Chloropseidae | 1 | 13 | 0.382152 | 1.589147 | 4.531902 |
| Ciconiidae | 3 | 29 | 0.21585 | 2.599555 | -1.38084 |
| Cinclidae | 1 | 6 | 0.318418 | 1.181141 | 3.019024 |
| Cinclosomatidae | 5 | 11 | 0.000967 | 1.430421 | -32.4507 |
| Cisticolidae | 6 | 161 | 0.012897 | 12.949 | 4.316149 |
| Climacteridae | 1 | 7 | 0.341424 | 1.284464 | 4.06377 |
| Columbidae | 17 | 349 | 0.005953 | 28.063 | 3.169914 |
| Coraciidae | 1 | 17 | 0.356567 | 1.776738 | 4.569047 |
| Corvidae | 19 | 136 | 0.007002 | 10.929 | -5.93456 |
| Cotingidae | 2 | 73 | 0.043018 | 5.986974 | 5.461608 |
| Cuculidae | 4 | 188 | 0.00039 | 15.33 | 6.026596 |
| Dicruridae | 2 | 30 | 0.26821 | 2.707371 | 2.357902 |
| Elanidae | 1 | 7 | 0.341424 | 1.285088 | 4.072682 |
| Emberizidae | 20 | 71 | 4.42E-07 | 5.744724 | -20.0779 |
| Estrildidae | 17 | 140 | 0.026501 | 11.321 | -4.05643 |
| Falconidae | 9 | 77 | 0.07733 | 6.18018 | -3.6621 |
| Falcunculidae | 2 | 3 | 0.018058 | 1.098131 | -30.0623 |
| Fringillidae | 26 | 252 | 0.037874 | 20.424 | -2.2127 |
| Furnariidae | 13 | 332 | 0.000979 | 26.682 | 4.121084 |
| Galbulidae | 1 | 19 | 0.336624 | 1.977415 | 5.144291 |
| Gaviidae | 1 | 10 | 0.378656 | 1.432337 | 4.323374 |
| Glareolidae | 6 | 24 | 0.008278 | 2.307955 | -15.3835 |
| Gruidae | 5 | 24 | 0.029687 | 2.160279 | -11.8322 |
| Haematopodidae | 2 | 6 | 0.070095 | 1.236715 | -12.7214 |
| Hirundinidae | 21 | 130 | 0.001042 | 10.544 | -8.04308 |
| Hypocoliidae | 1 | 2 | 0.148757 | 1.047337 | 2.366864 |
| Icteridae | 17 | 135 | 0.020942 | 10.735 | -4.64074 |
| Jacanidae | 3 | 9 | 0.026833 | 1.35514 | -18.2762 |
| Laniidae | 8 | 51 | 0.031102 | 4.174134 | -7.5017 |
| Laridae | 13 | 103 | 0.034378 | 8.452 | -4.41553 |
| Leiotrichidae | 6 | 140 | 0.032339 | 11.612 | 4.008571 |
| Locustellidae | 10 | 79 | 0.051365 | 6.373 | -4.59114 |
| Lybiidae | 1 | 53 | 0.053279 | 4.359596 | 6.33886 |
| Maluridae | 6 | 29 | 0.019157 | 2.513304 | -12.0231 |
| Meliphagidae | 12 | 192 | 0.073625 | 15.625 | 1.888021 |
| Meropidae | 1 | 41 | 0.113466 | 3.400624 | 5.855181 |
| Mimidae | 6 | 38 | 0.052094 | 3.195173 | -7.38112 |
| Mohouidae | 3 | 3 | 0.00053 | 1.084444 | -63.8519 |
| Monarchidae | 1 | 101 | 0.001768 | 8.24 | 7.168317 |
| Motacillidae | 11 | 86 | 0.042543 | 6.910911 | -4.75475 |
| Muscicapidae | 26 | 412 | 0.031284 | 33.259 | 1.761893 |
| Nectariniidae | 3 | 146 | 0.001546 | 11.951 | 6.130822 |
| Odontophoridae | 2 | 35 | 0.240575 | 2.990605 | 2.830301 |
| Oreoicidae | 1 | 3 | 0.205078 | 1.097222 | 3.240741 |
| Otididae | 5 | 31 | 0.065735 | 2.707212 | -7.39609 |
| Pachycephalidae | 1 | 50 | 0.064744 | 4.17915 | 6.3583 |
| Paradisaeidae | 1 | 43 | 0.10052 | 3.612308 | 6.075134 |
| Paradoxornithidae | 4 | 39 | 0.183979 | 3.340314 | -1.6915 |
| Pardalotidae | 1 | 5 | 0.288713 | 1.174927 | 3.498542 |
| Paridae | 8 | 63 | 0.068714 | 5.1167 | -4.57667 |
| Parulidae | 44 | 171 | 3.10E-12 | 13.855 | -17.6287 |
| Passerellidae | 42 | 172 | 5.58E-11 | 13.878 | -16.35 |
| Passeridae | 4 | 47 | 0.203112 | 3.897044 | -0.21906 |
| Pelecanidae | 1 | 11 | 0.382813 | 1.451827 | 4.10752 |
| Pellorneidae | 3 | 63 | 0.133101 | 5.063253 | 3.275005 |
| Petroicidae | 5 | 55 | 0.17758 | 4.44186 | -1.0148 |
| Phasianidae | 29 | 191 | 0.000425 | 15.602 | -7.01466 |
| Phylloscopidae | 14 | 128 | 0.05964 | 10.645 | -2.62109 |
| Picidae | 28 | 261 | 0.026102 | 21.077 | -2.65249 |
| Pittidae | 3 | 53 | 0.182588 | 4.29667 | 2.446547 |
| Ploceidae | 6 | 121 | 0.065842 | 9.654 | 3.019835 |
| Podicipedidae | 3 | 31 | 0.224291 | 2.598719 | -1.29445 |
| Pomatostomidae | 1 | 5 | 0.288713 | 1.194888 | 3.897764 |
| Prunellidae | 3 | 19 | 0.13311 | 1.909794 | -5.73793 |
| Psittacidae | 14 | 376 | 0.000283 | 30.182 | 4.303723 |
| Psophodidae | 1 | 5 | 0.288713 | 1.173228 | 3.464567 |
| Pteroclidae | 2 | 20 | 0.272422 | 2.01487 | 0.074349 |
| Ptilonorhynchidae | 4 | 20 | 0.053861 | 1.953431 | -10.2328 |
| Pycnonotidae | 5 | 159 | 0.00626 | 12.883 | 4.957862 |
| Rallidae | 20 | 166 | 0.020383 | 13.209 | -4.09096 |
| Recurvirostridae | 2 | 10 | 0.150039 | 1.411871 | -5.88129 |
| Regulidae | 3 | 10 | 0.035231 | 1.435764 | -15.6424 |
| Remizidae | 1 | 14 | 0.378243 | 1.61669 | 4.40493 |
| Rhinocryptidae | 3 | 60 | 0.147742 | 4.890452 | 3.150754 |
| Rhipiduridae | 3 | 63 | 0.133101 | 5.049296 | 3.25285 |
| Scolopacidae | 21 | 156 | 0.007202 | 12.592 | -5.38974 |
| Scotocercidae | 1 | 47 | 0.078393 | 3.865927 | 6.097718 |
| Sittidae | 5 | 34 | 0.083574 | 2.954879 | -6.01506 |
| Strigidae | 15 | 227 | 0.07416 | 18.34 | 1.471366 |
| Strigopidae | 2 | 3 | 0.018058 | 1.099174 | -30.0275 |
| Sturnidae | 16 | 129 | 0.026041 | 10.512 | -4.25426 |
| Sylviidae | 1 | 49 | 0.069036 | 3.983756 | 6.089299 |
| Thamnophilidae | 3 | 242 | 2.15E-06 | 19.517 | 6.825207 |
| Thraupidae | 21 | 423 | 0.00365 | 34.105 | 3.098109 |
| Threskiornithidae | 1 | 45 | 0.088858 | 3.673116 | 5.940258 |
| Timaliidae | 5 | 54 | 0.175652 | 4.438259 | -1.04026 |
| Tinamidae | 2 | 45 | 0.172133 | 3.711202 | 3.802672 |
| Trochilidae | 16 | 377 | 0.001147 | 30.784 | 3.921485 |
| Troglodytidae | 8 | 101 | 0.145162 | 8.153 | 0.151485 |
| Turdidae | 25 | 227 | 0.025898 | 18.223 | -2.98546 |
| Turnicidae | 4 | 20 | 0.053861 | 2.003606 | -9.98197 |
| Tyrannidae | 32 | 530 | 0.014098 | 42.993 | 2.074151 |
| Tytonidae | 1 | 16 | 0.365142 | 1.795918 | 4.97449 |
| Viduidae | 4 | 20 | 0.053861 | 2.012407 | -9.93797 |
| Vireonidae | 8 | 77 | 0.11455 | 6.154154 | -2.3972 |
| Zosteropidae | 4 | 122 | 0.017837 | 10.014 | 4.929508 |

Appendix S18. The difference between the number of AOH maps in each Red List category under-represented by KBAs (the 2190 AOH maps with <8% of their suitable habitat overlapping KBAs) and 1000 random samples of 2190 AOH maps from the full set of 11,985 terrestrial avian AOH maps, as a percentage of the total number of AOH maps in that category; i.e. the difference between observed under-represented species by KBAs and randomly selected species, by Red List category. A difference of 0 would mean the low overlap group is not different to the random samples, a negative difference would mean that category was less well represented than would be expected by chance, and a positive difference would mean that category was better represented than expected.

| Red List category | Number of AOH maps with under- representation | Total number of AOH maps | Chance that the number of AOH maps being under-represented is random | Mean number of AOH maps selected by chance (binomial distribution) | Percentage of total number of AOH maps difference between random samples and observed under-represented AOH maps |
| --- | --- | --- | --- | --- | --- |
| Critically endangered | 27 | 177 | 0.047262 | 32.135 | 2.90113 |
| Endangered | 56 | 386 | 0.008269 | 70.434 | 3.739378 |
| Vulnerable | 118 | 727 | 0.01411 | 132.793 | 2.034801 |
| Near threatened | 145 | 1003 | 0.000201 | 183.643 | 3.852742 |
| Least concern | 1822 | 9603 | 0.002163 | 1754.594 | -0.70193 |
| Data Deficient | 8 | 42 | 0.153775 | 7.649 | -0.83571 |


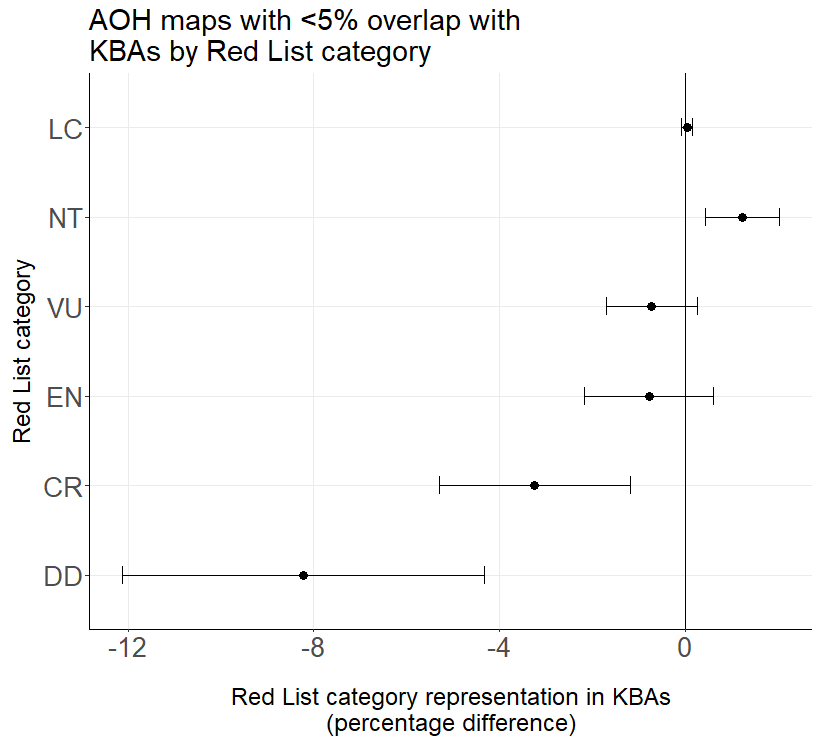


Poorly represented Well represented

Appendix S19. The difference between the number of AOH maps from each Red List category in the <5% overlap group (the 970 AOH maps with <5% of their suitable habitat overlapping KBAs) and 1000 samples of 970 AOH maps from the full set of 11,985 terrestrial avian AOH maps, as a percentage of the total number of AOH maps in that category. A difference of 0 would mean the low overlap group is not different to the random samples, a negative difference would mean that category was less well represented than would be randomly expected, and a positive difference would mean that category was better represented than randomly expected.

Appendix S20. The difference between the number of AOH maps in each Red List category very under-represented by KBAs (the 970AOH maps with <5% of their suitable habitat overlapping KBAs) and 1000 random samples of 970AOH maps from the full set of 11,985 terrestrial avian AOH maps, as a percentage of the total number of AOH maps in that category; i.e. the difference between observed under-represented species by KBAs and randomly selected species, by Red List category. A difference of 0 would mean the low overlap group is not different to the random samples, a negative difference would mean that category was less well represented than would be expected by chance, and a positive difference would mean that category was better represented than expected.

| Red List category | Number of AOH maps with under- representation | Total number of AOH maps | Chance that the number of AOH maps being under-represented is random | Mean number of AOH maps selected by chance (binomial distribution) | Percentage of total number of AOH maps difference between random samples and observed under-represented AOH maps |
| --- | --- | --- | --- | --- | --- |
| Critically endangered | 20 | 177 | 0.031395 | 14.271 | -3.23672 |
| Endangered | 34 | 386 | 0.062793 | 30.988 | -0.78031 |
| Vulnerable | 64 | 727 | 0.041006 | 58.757 | -0.72118 |
| Near threatened | 69 | 1003 | 0.017539 | 81.427 | 1.238983 |
| Least concern | 773 | 9603 | 0.014783 | 777.274 | 0.044507 |
| Data Deficient | 7 | 42 | 0.031984 | 3.547692 | -8.21978 |

Appendix S21. Area of Habitat (AOH) maps of threatened very-under-represented species, and the those with no overlap with KBAs, that do not overlap with protected areas.

| Species | Range | Red List category | Number of KBAs overlapping | Percentage of AOH in KBAs | Year recognized by BirdLife |
| --- | --- | --- | --- | --- | --- |
| *Erythropitta splendida* | Resident | VU | 0 | 0 | 2016 |
| *Scytalopus gettyae* | Resident | NT | 0 | 0 | 2016 |
| *Sitta insularis* | Resident | CR | 0 | 0 | 2016 |
| *Mixornis prillwitzi* | Resident | VU | 0 | 0 | 2016 |
| *Crossoptilon mantchuricum* | Resident | VU | 7 | 3.10 | 1988 |
| *Rhodonessa caryophyllacea* | Resident | CR | 0 | 0 | 1988 |
| *Numenius tenuirostris* | Breeding | CR | 0 | 0 | 1988 |
| *Rhinoptilus bitorquatus* | Resident | CR | 2 | 3.74 | 1988 |
| *Pseudorectes incertus* | Resident | NT | 0 | 0 | 1988 |
| *Rhipidura malaitae* | Resident | VU | 0 | 0 | 1988 |
| *Turdus feae* | Breeding | VU | 10 | 2.36 | 1988 |
| *Ficedula subrubra* | Breeding | VU | 3 | 0.60 | 1988 |
| *Chlorocichla prigoginei* | Resident | EN | 2 | 0.86 | 1988 |
| *Mirafra sharpii* | Resident | EN | 2 | 0.90 | 1988 |
| *Amandava formosa* | Resident | VU | 50 | 2.94 | 1988 |
| *Emberiza jankowskii* | Resident | EN | 7 | 3.76 | 1988 |
| *Atlapetes pallidiceps* | Resident | EN | 0 | 0 | 1988 |
| *Aegotheles tatei* | Resident | DD | 0 | 0 | 2000 |
| *Lipaugus weberi* | Resident | CR | 0 | 0 | 2002 |
| *Spelaeornis chocolatinus* | Resident | VU | 2 | 2.37 | 2008 |
| *Phapitreron frontalis* | Resident | CR | 0 | 0 | 2014 |

# Supporting information references

Bell, D. (2019) *Hurricane Dorian was also a catastrophe for the Bahamas’ unique birds*, *The Conversation*. Available at: https://theconversation.com/hurricane-dorian-was-also-a-catastrophe-for-the-bahamas-unique-birds-123493 (Accessed: 1 June 2023).

BirdLife International (2023) *Slender-billed Curlew (Numenius tenuirostris) - BirdLife species factsheet*. Available at: http://datazone.birdlife.org/species/factsheet/slender-billed-curlew-numenius-tenuirostris/text (Accessed: 3 October 2023).

Cuervo, A.M. (2014) ‘Lipaugus weberi’, in *Libro rojo de aves de Colombia, Volumen I: bosques húmedos de los Andes y la costa Pacífica*. Bogota, pp. 268–270.

De Camargo, C., Gibbs, H.L., Costa, M.C., Del-Rio, G., Silveira, L.F., Wasko, A.P. and Francisco, M.R. (2015) ‘Marshes as “Mountain Tops”: Genetic Analyses of the Critically Endangered São Paulo Marsh Antwren (Aves: Thamnophilidae)’, *PLOS ONE*, 10(10), p. e0140145. Available at: <https://doi.org/10.1371/JOURNAL.PONE.0140145>.

Parks, S. (2023) *The Last Days of the Bahama Nuthatch - Atlas Obscura*, *Atlas Obscura*. Available at: https://www.atlasobscura.com/articles/bahama-nuthatch-extinction (Accessed: 1 June 2023).

Pereira, D.J., Gardner, M.A., Geary, M., Bell, D.J. and Collar, N.J. (2023) ‘Distribution and habitat requirements of the Bahama Warbler Setophaga flavescens on Grand Bahama in 2018’, *Bird Conservation International*, 33, p. e46. Available at: <https://doi.org/10.1017/S095927092200048X>.
